# Tumor targeting with pH-responsive poly(2-oxazoline)-based nanogels for metronomic doxorubicin treatment

**DOI:** 10.18632/oncotarget.24806

**Published:** 2018-04-27

**Authors:** Doerte Hoelzer, Meike N. Leiske, Matthias Hartlieb, Tanja Bus, David Pretzel, Stephanie Hoeppener, Kristian Kempe, René Thierbach, Ulrich S. Schubert

**Affiliations:** ^1^ Institute of Nutrition, Friedrich Schiller University Jena, 07743 Jena, Germany; ^2^ Laboratory of Organic and Macromolecular Chemistry (IOMC), Friedrich Schiller University Jena, 07743 Jena, Germany; ^3^ Jena Center for Soft Matter (JCSM), Friedrich Schiller University Jena, 07743 Jena, Germany; ^4^ Current address: Institute of Biomaterial Science, Helmholtz-Zentrum Geesthacht, 14513 Teltow, Germany; ^5^ Current address: Monash Institute of Pharmaceutical Sciences, Monash University, Parkville, VIC 3052, Australia

**Keywords:** poly(2-oxazoline), doxorubicin, drug delivery, nanogel, metronomic

## Abstract

The synthesis of a new nanogel drug carrier system loaded with the anti-cancer drug doxorubicin (DOX) is presented. Poly(2-oxazoline) (POx) based nanogels from block copolymer micelles were cross-linked and covalently loaded with DOX using pH-sensitive Schiff’ base chemistry. DOX loaded POx based nanogels showed a toxicity profile comparable to the free drug, while unloaded drug carriers showed no toxicity. Hemolytic activity and erythrocyte aggregation of the drug delivery system was found to be low and cellular uptake was investigated by flow cytometry and fluorescence microscopy. While the amount of internalized drug was enhanced when incorporated into a nanogel, the release of the drug into the nucleus was delayed. For *in vivo* investigations the nanogel drug delivery system was combined with a metronomic treatment of DOX. Low doses of free DOX were compared to equivalent DOX loaded nanogels in a xenograft mouse model. Treatment with POx based nanogels revealed a significant tumor growth inhibition and increase in survival time, while pure DOX alone had no effect on tumor progression. The biodistribution was investigated by microscopy of organs of mice and revealed a predominant localization of DOX within tumorous tissue. Thus, the POx based nanogel system revealed a therapeutic efficiency despite the low DOX concentrations and could be a promising strategy to control tumor growth with fewer side effects.

## INTRODUCTION

In modern oncology it is a major challenge to deliver therapeutic agents more safely and directly to the tumor. Doxorubicin (DOX) is an anthracycline antibiotic and is one of the most effective as well as commonly used chemotherapeutic drugs. It is used as a first-line treatment of various types of cancer, including hematologic malignancies, breast and ovarian carcinoma, neuroblastoma as well as soft tissue and bone sarcoma. The antitumor activity of DOX can be triggered by different mechanisms: (i) By intercalating into DNA strands and (ii) prevention of replication and transcription of DNA by inhibiting the enzyme topoisomerase II or, (iii) formation of free radicals leading to membrane and DNA damage as well as apoptosis [1, 2] However, the clinical benefit of DOX is limited by different side effects, *i.e.* cardiotoxicity [3, 4].

The use of nanosized drug carriers is rapidly emerging and can help to reduce these side effects as well as improve the drugs solubility [5], blood circulation time [6] and tissue distribution [7]. In particular nanogels, hydrogel nanoparticles with crosslinked hydrophilic polymers, offer several advantages for their use as a drug delivery system [8]. For this reason, the utilization of nanocarriers (*e.g.* nanoparticles) in terms of delivery of anti-cancer drugs has increased significantly during the last years [9–11]. Nanogels enable a high drug loading capacity, can protect and shield drugs until they reach their desired target and are highly biocompatible and biodegradable [12]. Due to the leaky structure of cancerous tissue together with the lack of effective lymphatic drainage, nanogels tend to accumulate in the tumor tissue known as enhanced permeability and retention (EPR) effect [13]. To achieve an effective delivery of the drug to the tumor it is also very important to prevent a premature disassembly or drug release from the carrier. A common strategy is the use of covalently cross-linked drug delivery systems (*i.e.* core cross-linked micelles or other nanogels) and a likewise covalently but reversibly attached drug [14–16].

The majority of drug delivery systems utilize a poly(ethylene glycol) (PEG) shell to shield themselves from unspecific interactions with healthy tissue or the components of the blood stream. However, reports about complement activation by PEG [17–19] and vacuolation [20–22] in the body have raised concerns about safety and reliability of the polymer. Poly(2-oxazoline)s (POx) represent a promising alternative as they are biocompatible, [13, 23, 24] and show a stealth behavior similar to PEG when the side chain substitution is chosen correctly [25, 26]. Recent studies elucidate the pharmacokinetic behavior of the polymer dependent on its molar mass, demonstrating superior behavior when compared to PEG [27, 28]. The first clinical study using a POx derivative is currently ongoing (SER-214, phase I) [14] and the polymer was approved by the federal food administration (FDA) as an indirect additive used in food contact substances (21CFR175.105) in 2016. In addition, POx based formulations of the cancer drug paclitaxel show great promise *in vivo* [5]. One major advantage of the polymer over PEG is its versatile functionalization chemistry [29] enabling easy access to a multitude of functional polymers and materials [15]. POx based nanogels have been reported, [30] but far have not been exploited for the use as a cancer drug delivery system.

Recently, we reported the synthesis of nanogels based on double hydrophilic POx block copolymers. They were based on micellar architecture with a cationic block forming the core and a poly(2-ethyl-2-oxazoline)(P(EtOx)) shell. The material was cross-linked and dye loaded by imine bonds [31]. The materials showed excellent biocompatibility and their charge and cellular uptake could be tailored by varying the cross-linking density [32]. Within this contribution, DOX is to be used as a payload in order to increase the efficiency and specificity of the drug towards cancer cells *in vitro* and *in vivo*. Drug attachment as well as cross-linking is accomplished using pH sensitive Schiff's base chemistry, to enable intracellular drug release [31, 33].

In addition to the drug delivery system itself, the regime of drug administration is of particular interest. Conventional chemotherapy relies on the administration of the maximum tolerated dose (MTD) to achieve the desired effect without unacceptable side effects. Because of the high toxicity and potential development of chemoresistance other concepts of drug administration are evolving. Metronomic chemotherapy is defined as a chronic administration of low doses of cytotoxic agents and can help to improve the efficiency of cancer treatment [34, 35]. Herein we report the straightforward synthesis of a POx based nanogel in a one pot approach, reversibly linked to (or loaded with) the anti-cancer drug DOX. The drug delivery system is biocompatible and able to release its payload as shown by *in vitro* investigations. In addition, *in vivo* experiments in mice show a promising increase in survival rate as compared to pure DOX at relatively low concentrations.

## RESULTS AND DISCUSSION

### Synthesis and loading of the poly(2-oxazoline)-based nanogels

Polymers were synthesized by sequential monomer addition using microwave technology employing 2-ethyl-2-oxazoline (EtOx) for the first and 2-(4-((*tert*-butoxycarbonyl)amino)butyl)-2-oxazoline (BocOx) for the second block. The second monomer was introduced within a glove box under nitrogen atmosphere to reduce termination prior to block extension. P(EtOx_98_-*b*-BocOx_32_) (1) was synthesized with a narrow dispersity of Ð = 1.07, which did not increase drastically after deprotection of the amine groups to yield poly(2-ethyl-2-oxazoline)-*block*-(poly(2-(4-amino)butyl)-2-oxazoline)) (P(EtOx-*b*-AmOx)) (Supplementary Table 1, Supplementary Scheme 1, Supplementary Figures 1–2). While size exclusion chromatography (SEC) measurements of initial polymers could be performed in chloroform, deprotected P(EtOx_98_-*b*-AmOx_32_) (2) had to be measured in *N*,*N*-dimethylacetamide (DMAc), explaining the difference in molar mass compared to the precursor polymer.

While DOX is fluorescent and can, therefore, be tracked directly within cells, its emission is highly dependent on the environment [36, 37]. To circumvent this issue and create nanogels, which can be tracked independent of their DOX release, polymer 2 was labeled with a fluorescent dye prior to the nanogel preparation. To this end, a dye with a near-infra red fluorescence (Alexafluor 660) was chosen to not interfere with the fluorescence of the drug. The dye possesses a *N*-hydroxy succinimide (NHS) ester function, able to react with the amine groups of the P(AmOx) block of polymer 2. One equivalent of dye per polymer chain was applied to retain a sufficient amount of free amine groups for further self-assembly processes, and cross-linking reactions. To separate the labeled polymer 3 from unreacted dye molecules, precipitation in diethyl ether, as well as dialysis in deionized water was performed. The success of the attachment was confirmed by SEC measurements (Supplementary Figure 2) comparing the refractive index (RI). and UV traces of the polymer. The lack of an UV signal at high elution volumes indicates the absence of unbound dye. The fluorescence maximum of the dye coupled to the polymer was found to be similar to the free chromophore (Supplementary Figure 3). The coupling efficiency as determined by the emission of the polymer was determined *via* UV/Vis measurements and found to be 30%.

**Figure 1:**
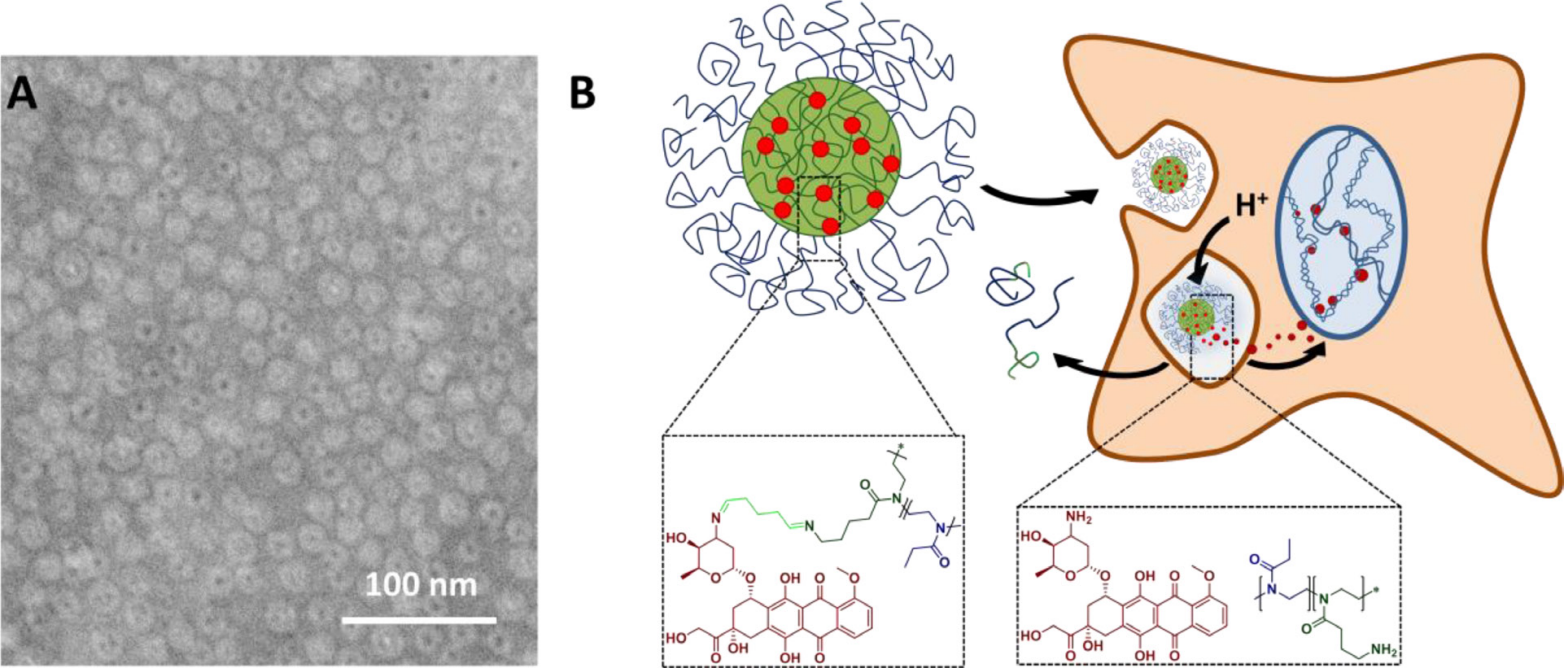
(**A**) Schematic representation of nanogels obtained from P(EtOx_98_-*b*-BocOx_32_) with a P(AmOx) core loaded with DOX and a P(EtOx) shell, cryo-TEM image of unlabeled DOX-nanogel in water (scale bar represents 100 nm), and (**B**) a schematic depiction of the drug delivery route of DOX

The self-assembly of these systems to form polymeric micelles was conducted as reported previously [31]. Briefly, the polymers were dissolved in chloroform, which leads to the formation of micellar structures comprising a P(AmOx) core and a P(EtOx) shell. Cross-linking was performed using glutaraldehyde (GA) resulting in the formation of nanogels. As previous investigations [32] showed a reduced cellular uptake of systems with a higher cross linking density, three equivalents of cross-linker (in respect to amine groups) were used. A reduced positive charge density is supposed to lead to prolonged circulation times *in vivo*. Drug loading was performed by reacting excessive aldehyde functionalities with DOX. The free amine groups of the molecule reacts with free aldehyde groups of the cross-linker resulting in a covalent attachment to the nanogel (Figure 1). As the imine function, which stabilizes the nanogel core and the drug, is labile at low pH values, created systems are expected to be disintegrating. Alexafluor-labeled (referred to as “labeled DOX-nanogel”), as well as unlabeled DOX-containing (referred to as “unlabeled DOX-nanogel”) nanogels were produced. As a non-toxic equivalent DOX-free 6-aminofluorescein (6AF) loaded nanogels (referred to as “DOX-free nanogel”) were synthesized using the same method.

### Characterization of the nanogels

Due to the fact that the drug is not encapsulated into, but covalently bound to the nanogel, the term loading efficiency is used instead of the commonly utilized encapsulation efficiency. To determine the loading efficiency of produced nanogels the absorbance of the system was measured and compared to a calibration of the small molecule (Supplementary Figure 4). In the case of DOX-free nanogels, absorbance was measured at λ_ex_ = 490 nm whereas for labeled and unlabeled DOX-nanogels the absorbance was detected at λ_ex_ = 480 nm. A three-fold difference in mass loading was observed between fluorescein (17 wt%) and DOX (5 to 6 wt%) immobilization, (Table 1), while loading was relatively independent on the presence of Alexafluor 660 labels on the polymer chain. The difference can be explained by the nature of the cargo molecules. While both possess an amine functionality, which can be coupled covalently to aldehyde functionalities, 6AF also possesses a carboxylic acid function, which can interact in an electrostatic way with the positively charged core of the nanogel, leading to an increase in loading efficiency by electrostatic interaction.

**Table 1:** Analytical data of nanogels formed by the self-assembly of polymers 2 and 3

Sample	Precursor polymer	Capping agent	Size (DLS) [d, nm]	ζ [mV]	Content of capping agent [wt%]	Size (cryoTEM) [d, nm]
**DOX-free** **nanogel**	2	6AF	24	7	17	15
**Unlabeled** **DOX-nanogel**	2	DOX	26	18	5	20
**Labeled** **DOX-nanogel**	3	DOX	15	25	6	15

DLS and zeta potential values are determined in water. Sizes determined by DLS are derived from the number distribution.

As shown in Supplementary Figure 4, the fluorescence spectrum of DOX broadens significantly when incorporated into nanogels. The emission properties of the chromophore are known to be highly dependent on environmental factors [36, 37]. The presence of amine groups within the core of the nanogel and other factors are likely to influence the fluorescence of DOX. In the case of the Alexafluor 660 labeled systems a high wavelength shoulder is visible in the emission spectrum indicating the presence of the near-IR dye. Upon excitation at λ_ex_ = 600 nm a pronounced fluorescence with a maximum at λ_em_ = 675 nm can be observed (Supplementary Figure 5).

To visualize the synthesized nanostructures, cryoTEM measurements were performed (Figure 1, Supplementary Figure 6). The images showed monodisperse spherical structures for all samples. For DOX-free and labeled DOX-nanogels an average diameter of 15 nm was obtained while the diameter of unlabeled DOX-nanogels was found to be 20 nm (Table 1, Supplementary Figure 6B and 6C). In addition, in the case of unlabeled DOX-nanogels a core-shell structure could be visualized showing a dark center and a lighter corona. The core is likely to be compact in water due to the presence of the hydrophobic DOX, whereas the shell is water swollen resulting in a lower contrast. For DOX-free nanogels and labeled DOX-nanogels this structure could not be visualized, which is possibly a result of the dense packing of nanostructures on the TEM grid. If the P(EtOx) shell is partially not visible due to overlap and lacking contrast this could explain the size discrepancy between the nanogels. The size was confirmed by dynamic light scattering (DLS) measurements (Table 1). Zeta potential measurements show positive values for all nanogels, which was expected due to the cationic nature of the micellar core. Fluorescein loaded nanogels show a lower zeta potential as compared to DOX loaded samples, which can be explained by the compensation of cationic charges by the anionic nature of fluorescein. This finding is in line with the increased loading of fluorescein quenched nanogels as compared to structures with DOX as a cargo.

The most important requirement for a drug carrier is the site specific release of the drug. As cargo molecules within the produced nanogels are attached *via* imine bonds, which are known to be reversible at pH values below 7, [38] a release within endosomal or lysosomal cellular compartments is likely as previously shown by M. Hruby and co-workers [39]. In order to investigate the stability of the nanogels at 4°C (storage temperature) and 37°C (human body temperature) at a pH value of 7.4, the z-average and the polydispersity index (PDI) as well as the number mean size value of the nanogels was determined using DLS measurements (Supplementary Figure 7). Nanogels were determined to be stable during the entire measurement time of two weeks, revealing no significant changes in size or PDI. Furthermore, it was necessary to determine the possibility of a drug release at a lysosomal pH value of 5. J. S. Basuki *et al.* previously investigated iron oxide nanoparticles that were loaded with DOX *via* pH sensitive imine bond *via* DLS measurements, revealing an increase in the particle size at a pH value of 5, caused by drug release [40]. Since glycine was determined to be essential for cancer cell proliferation and, consequently, is present within tumorous compartments, [41] DLS investigations of the labeled DOX-nanogels were conducted in phosphate buffered saline (PBS) and glycine was added representing a competitive amine to the imine bond (Supplementary Figure 8). While labeled DOX-nanogels did not reveal significant changes in size or PDI at a pH value of 7.4, both increase at a pH value of 5.0. Herein, it is noteworthy that after a second addition of glycine, this trend further increases. This might be beneficial for triggering the endosomal burst, as recently shown in gene transfection applications within our group [42]. In order to obtain additional qualitative information about the release of DOX from the labeled DOX-nanogel, diffusion order spectroscopy (DOSY) NMR measurements were also conducted (Supplementary Figures 9 and 10). Hereby, the diffusion coefficients of labeled DOX-nanogels in NaCl were compared to labeled DOX-nanogels in 150 mM PBS (pH = 5.0), which contained glycine. Pure DOX and glycine were evaluated for comparison. A stacking of the spectra suggests the release of DOX at pH 5.0, while no DOX release could be determined in NaCl (Supplementary Figure 9). Unfortunately, a quantification of the DOX release from the labeled DOX-nanogels was not possible by the applied methods.

### *In vitro* cytotoxicity of nanogels

**Figure 2:**
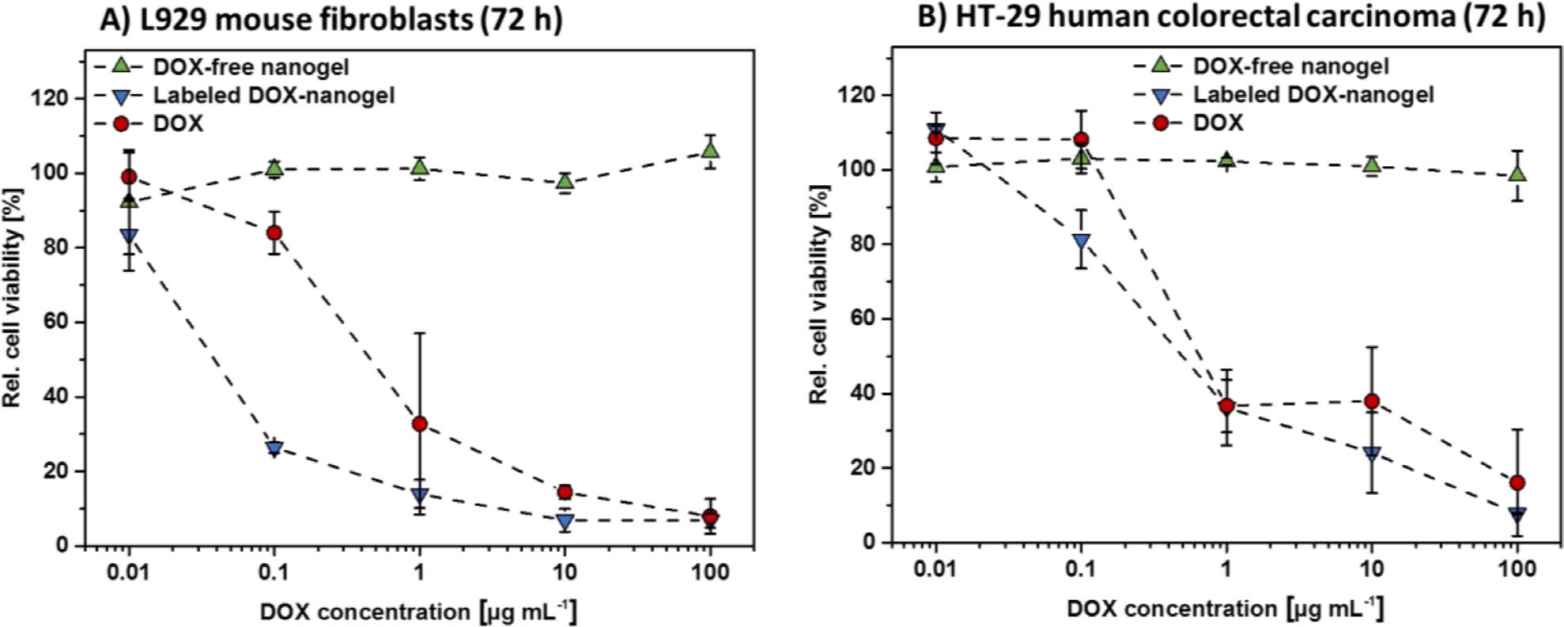
Cytotoxicity of DOX-free nanogels, labeled DOX-nanogels as well as free DOX were determined by XTT assay L292 mouse fibroblasts (**A**) as well as HT-29 human colorectal carcinoma cells (**B**) were incubated for 72 h with testing substances. DOX-nanogels were used at a concentration where the amount of loaded drug resembles the amount of DOX used per data point (polymer concentration 17 times higher than DOX concentration). DOX-free nanogels were used at the same polymer concentration as DOX-nanogels. Data are expressed as mean ± SD of six determinations.

One major mechanism of DOX is the intercalation into the minor groove of DNA [3]. Therefore, the molecule must penetrate the barrier of the nucleus to take effect. In order to verify whether DOX loaded nanogels are able to release DOX within cells, the cytotoxicity of labeled DOX-nanogels in comparison to free DOX and DOX-free nanogels was investigated (Figure 2, Supplementary Figure 11). The influence of the materials on the cell viability was probed using two different cell lines. L292 mouse fibroblasts are known to be sensitive to cytotoxic substances [43] and are used in the general assessment of biocompatibility (ISO 10993-5). Cytotoxicity tests were also performed with the human colorectal cancer cells HT-29, due to their ability to form tumors in nude mice and their usage for the nanogel *in vivo* studies that are described in later sections.

Cells were treated with nanogels or pure drug at varying concentrations for 24 h (Supplementary Figure 11) and 72 h (Figure 2), respectively. The amount of DOX loaded nanogels was chosen, so that the concentration of cargo drug matches the concentration of the free drug used for the tests. The concentration of DOX-free nanogels used, was identical to its DOX carrying equivalent in order to investigate the influence of the bare drug delivery system. DOX-free nanogels showed no adverse effects on both cell lines independent of incubation time or concentration. This was expected as P(EtOx) is considered to be biocompatible [13, 23, 24] and proves that neither cationic charges, nor potentially released 6AF influence the metabolism of the cells in a negative way. In contrast free DOX, as well as labeled DOX-nanogels, both show a time- and concentration-dependent decrease in cell viability for both cell lines. The effect is more pronounced for L929 mouse fibroblasts as they are more sensitive to cytotoxic effects. A 72 h treatment of L929 cells (Figure 2A) with labeled DOX-nanogels showed an increased cytotoxicity revealing an IC_50_ value of 0.043 μg mL^–1^ compared to a 24 h treatment (Supplementary Figure 11). Cytotoxicity of pure DOX was found to be lower, with an IC_50_ value of 0.547 μg mL^–1^ (72 h). This might be attributed to an enhanced internalization of the nanogels compared to the free drug [44]. For HT-29 cells (Figure 2B) this difference is less pronounced, with IC_50_ values of labeled DOX-nanogels of 0.752 μg mL^–1^ and pure DOX of 1.998 μg mL^–1^, respectively. From the reduced viability of cells a release of DOX from the nanogels can be assumed, which is essential for the known toxic effect of the drug.

### Cellular uptake and biocompatibility *in vitro*

**Figure 3:**
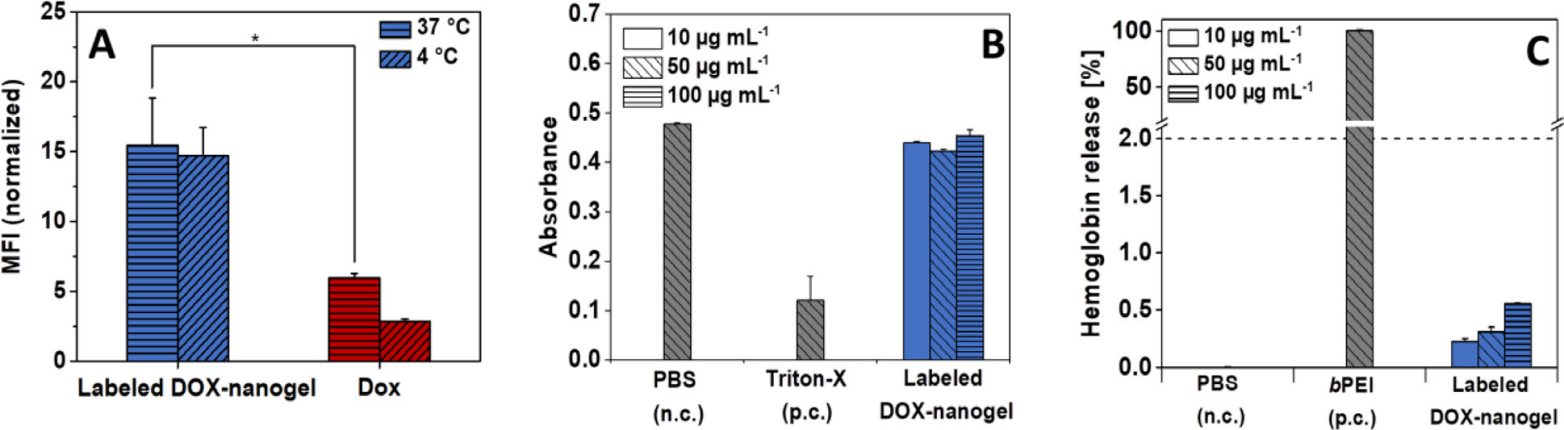
(**A**) Cellular uptake of DOX and labeled DOX-nanogels into HT-29 cells (0.01 mg mL^–1^) in dependence on the incubation time and temperature. Statistical differences are displayed as ^*^p < 0.05 and according to a Student's *t*-test. For amount of fluorescent cells see Supplementary Figure 12. (**B**) Erythrocyte aggregation of DOX-nanogels compared to PBS (negative control) and branched poly(ethylene imine) (positive control) using sheep blood of three different donors. (**C**) Hemolytic activity of DOX-nanogels compared to PBS (negative control) and *b*PEI (positive control) using sheep blood of three different donors.

To investigate whether the improved performance of nanogels is a result of an enhanced cellular uptake, flow cytometry measurements were performed after incubation with labeled DOX-nanogels and free DOX (Figure 3A and Supplementary Figure 12). HT-29 cells were used for the experiments as an *in vitro* cancer model, which was later used for xenograft mouse experiments. The fluorescence of DOX was quantified to determine the amount of DOX internalized within the cells. To elucidate the nature of uptake (energy dependent *vs.* energy independent) the experiments were performed at 37°C and 4°C [45], respectively. For an energy dependent uptake, a significant decrease of the amount of internalized drug would be expected as the metabolism of cells at 4°C is considerably slowed down. Incubation of HT-29 cells with labeled DOX-nanogels or pure DOX at 4°C reduced the cellular uptake compared to an incubation at 37°C. Therefore, cellular uptake seems to be energy-dependent, which would suggest an uptake by endocytosis. Additionally, cells treated with labeled DOX-nanogels possessed a higher fluorescence signal after 24 h treatment at 37°C compared to DOX alone. These findings suggest a higher accumulation of the nanogels in the cells [44, 46] caused by a P-glycoprotein mediated efflux of the pure drug, mostly known from multi drug resistant breast cancer cells [47, 48].

Besides cellular uptake and intracellular drug release, a further requirement of a drug carrier that strives to target cancerous tissue by *i.e.* the EPR effect is a low level of unspecific interaction with *i.e*. healthy tissue or the components of the blood stream. For this reason P(EtOx) was chosen as a shell material as it is well-known that P(EtOx) exhibits stealth properties and shows a blood circulation behavior *in vivo* similar to PEG [27] One prerequisite for a prolonged circulation in the blood stream is the hemocompatibility of the compound comprising the absence of blood clotting as well as lysis of red blood cells. The biocompatibility of labeled DOX-nanogels was tested against sheep blood (Figure 3B and 3C). The compound induced no major aggregation of red blood cells as compared to the positive control branched poly(ethylene imine) (*b*PEI) (25 kDa) as demonstrated in Figure 3B. While the high cationic charge density of PEI results in blood clotting, the cationic charges of the nanogels are shielded within the core of the structure and cannot directly lead to a precipitation of erythrocytes. Also, hemolysis as measured by the absorbance intensity in the blood plasma caused by leakage of hemoglobin release from red blood cells supports the biocompatibility of the drug carrier. While nanogels show slight hemoglobin release of erythrocytes, the total amount as compared to the surfactant Triton-X100 which served as a positive control is well below 2%, which is generally considered as a threshold for hemolytic activity (according to the ASTM F756-00 standard).

**Figure 4:**
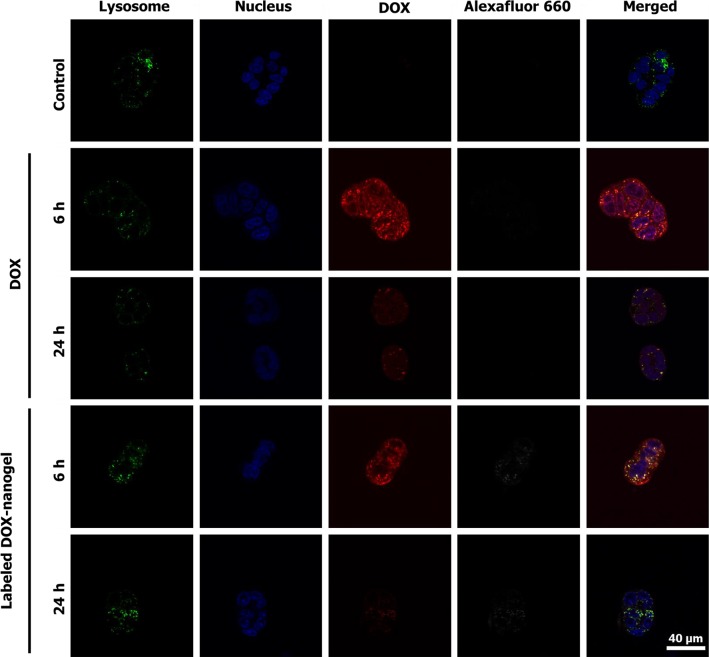
CLSM images of free DOX and labeled DOX-nanogels incubated with HT-29 colorectal carcinoma for 6 h or 24 h Lysosomal cellular compartments were stained green using LysoTracker Green DND-26 and the nucleus was labeled with Hoechst 33342 (blue). The fluorescence of DOX is depicted in red and the Alexafluor label of the polymer is shown in white.

To elucidate the uptake and intracellular activity of the nanogels further, their intracellular localization in L929 (Supplementary Figure 13) and HT-29 cells (Figure 4) was investigated using confocal laser scanning microscopy (CLSM). The nucleus was stained using Hoechst 33342 in order to examine its colocalization with DOX, which is indicative for release and activity of the drug. Lysosomal cellular compartments were stained using LysoTracker Green DND-26 and DOX was monitored *via* its fluorescence between λ_em_ = 600 to 650 nm. In addition, the polymer was tracked using the attached Alexafluor label measuring the emission between λ_em_ = 725 to 800 nm (Supplementary Figure 14). A first measurement was conducted after 6 h (Figure 4). Free DOX mainly shows a diffuse localization in the cytosol but is also to a certain extend present in the nucleus. Previous studies already reported a successful uptake and nucleus co-localization of DOX after 3 h incubation time, while the drug in polymersomes exhibited significantly longer times to enter the cell nucleus [46, 49]. In contrast, DOX-nanogels do not show a colocalization with the staining of the nucleus. For the labeled DOX- nanogels the overlap between red and green channel as well as the dotted structure of the signal suggests a lysosomal localization, which indicates an endocytic uptake mechanism [50] The presence of a polymer signal at the same position indicates that these signals represent intact nanogels that have not yet released the drug or been degraded.

Previous studies within our group already showed slower drug accumulation of the drug within the nucleus when using polymeric nanoparticles as drug delivery scaffolds [51]. For this reason, a second set of images was taken after 24 h incubation. After this time, the free drug is mostly localized in the nucleus of the cell. It can be assumed that DOX has either intercalated into the DNA in the nucleus or was excreted by the cells. However, also in the case of labeled DOX-nanogels a release of DOX into the nucleus was observed. The, in comparison to the DOX fluorescence, faint signal of the polymer suggests a partial degradation of the micelles. In addition, the signal is mostly associated with an extra nuclear localization. For longer incubation times it was increasingly difficult to locate intact cells for imaging due to the toxicity of the drug loaded system.

These results suggest that the uptake of nanogels is partially realized through endocytosis and that the material is degraded intracellularly, which leads to a release of the drug. Toxicity levels of the drug delivery system as well as co-localization studies indicate an accumulation of DOX in the nucleus after delivery to the cell. The kinetic as compared to the free drug is markedly slowed, which is probably associated to the release kinetic from the nanogel. An additional reason could be found in the dependence of the fluorescence of DOX on its environment. It is reported that the fluorescence signal of the drug strongly decreases upon intercalation with genetic material [36] and can be increased by incorporation in membranes or micelles [37]. Consequently, in the case of DOX associated nanogels, the fluorescence of the drug carrier in the cytosol is likely to outshine the intercalated drug. The similar toxicity of both, the free drug and the nanogel, however, suggests an efficient uptake and release of DOX within the cell.

### *In vivo* biocompatibility and biodistribution

The conclusion that can be drawn from the *in vitro* results is that DOX loaded nanogels are relatively stable outside cells but will release the drug once taken up into the endosome, which can later on fuse with a lysosome due to pH sensitivity in acidic compartments. Furthermore, they represent ideal candidates to exploit the EPR effect, since they reveal optimal sizes of approximately 20 nm in diameter as well as the P(EtOx) shell, which will shield them to a certain extend from unspecific interactions. To test this hypothesis *in vivo* studies on male athymic nude mice (Crl:CD1-Foxn1^nu^) with HT-29 originated tumors were conducted. In comparison to other studies with DOX loaded drug delivery systems [39] a relatively low DOX concentration was used in line with the concept of metronomic chemotherapy. In a first stage of the investigation the general biocompatibility was probed. Tumor-free nude mice were injected *via* tail vein with a single dose of labeled DOX-nanogels (corresponding to a DOX concentration of 0.3 or 1 mg kg^–1^) or with the same volume of the 0.9 wt% NaCl solution as the negative control. Body weight was monitored for 2 weeks (Supplementary Figure 15). As expected, no negative influence on the development of body weight was detected and no obvious signs of toxicity (changes in physical activity or constitution) were observed for these low DOX concentrations. For further analysis the 1 mg kg^–1^ DOX concentration was chosen.

**Figure 5:**
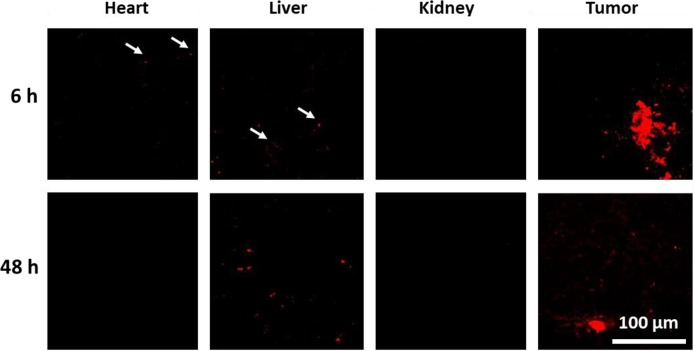
Confocal fluorescence images of histological samples derived from organs of mice that were treated with labeled DOX-nanogels at 1 mg kg^-1^ Fluorescence of DOX is shown in red. See Supplementary Figure 16 for control sample and 72 h labeled DOX-nanogel sample. See Supplementary Figure 17 for transmitted light images.

It is already known that the biodistribution of drugs can be influenced by polymeric drug carriers, [52] *i.e.* when equipped with targeting units [53, 54]. Furthermore, the blood clearance and organ accumulation rates of POx-DOX conjugates were determined to be advantageous for cancer therapy, as the conjugates express high blood circulation times of more than 24 h (t_1/2_ DOX = 4 min [55]) and tumor accumulation [39]. For this reason, the biodistribution of the drug carrier within the body was investigated using male nude mice, which received a subcutaneous injection of HT-29 cells (1 × 10^6^ cells in 250 μL) into the flank. When the tumor reached 6 to 8 mm, mice were treated with a single dose of either labeled DOX-nanogels at 1 mg kg^–1^ (150 μl) or of a NaCl solution with the same volume. The mice were sacrificed after predetermined time points (6, 48, and 72 h) and several organs (heart, liver, and kidney) as well as the tumor were excised and prepared for cryo-sections. Sections of mentioned organs were cut to a thickness of 8 μm and embedded in a water-based mounting medium on glass slides. The obtained samples were investigated by CLSM in order to monitor the accumulation of DOX in different body compartments. Histological samples of the tumor clearly show an accumulation of DOX as evident by the inhomogeneous red fluorescence (Figure 5, Supplementary Figure 16). The fluorescence signal is most pronounced 6 h after the injection and is still detectable after 48 h, but to a lesser extent. This phenomenon is also known from other studies. M. Hruby and coworkers determined the radioactive intensity of a ^125^I-labeled DOX carrier. Here, the mean radioactive intensity decreases significantly between 24 h and 72 h. Furthermore, the main amount of the carrier remains within the blood [39]. Since we used a comparable polymer system, a similar pharmacokinetic behavior might be favorable. Traces of DOX could also be observed in the liver in the form of small aggregates of about 1 μm size. The number of these aggregates increases over time, which points into the direction of either an accumulation in liver tissue or an excretion *via* the organ. Previously, a diminished accumulation of DOX loaded glycolchitosan nanoparticles within the heart could be determined [52]. Also in our study, only minor traces of DOX could be detected in the heart, which is promising, as cardiotoxicity is the most common side effect of DOX. No signal could be detected in the kidney indicating either a fast renal clearance of the nanogels or, more probably, no involvement of the kidney on the excretion of the nanogels. Small polymer-drug conjugates and nanoparticles with an average size below 5 nm are preferably renal excreted [56] and consequently accumulate within the kidney [39]. However, the utilized nanogels within this study possess an average diameter of around 20 nm and for this reason, an accumulation within the liver is more likely [56].

### *In vivo* anti-tumor efficiency

**Figure 6:**
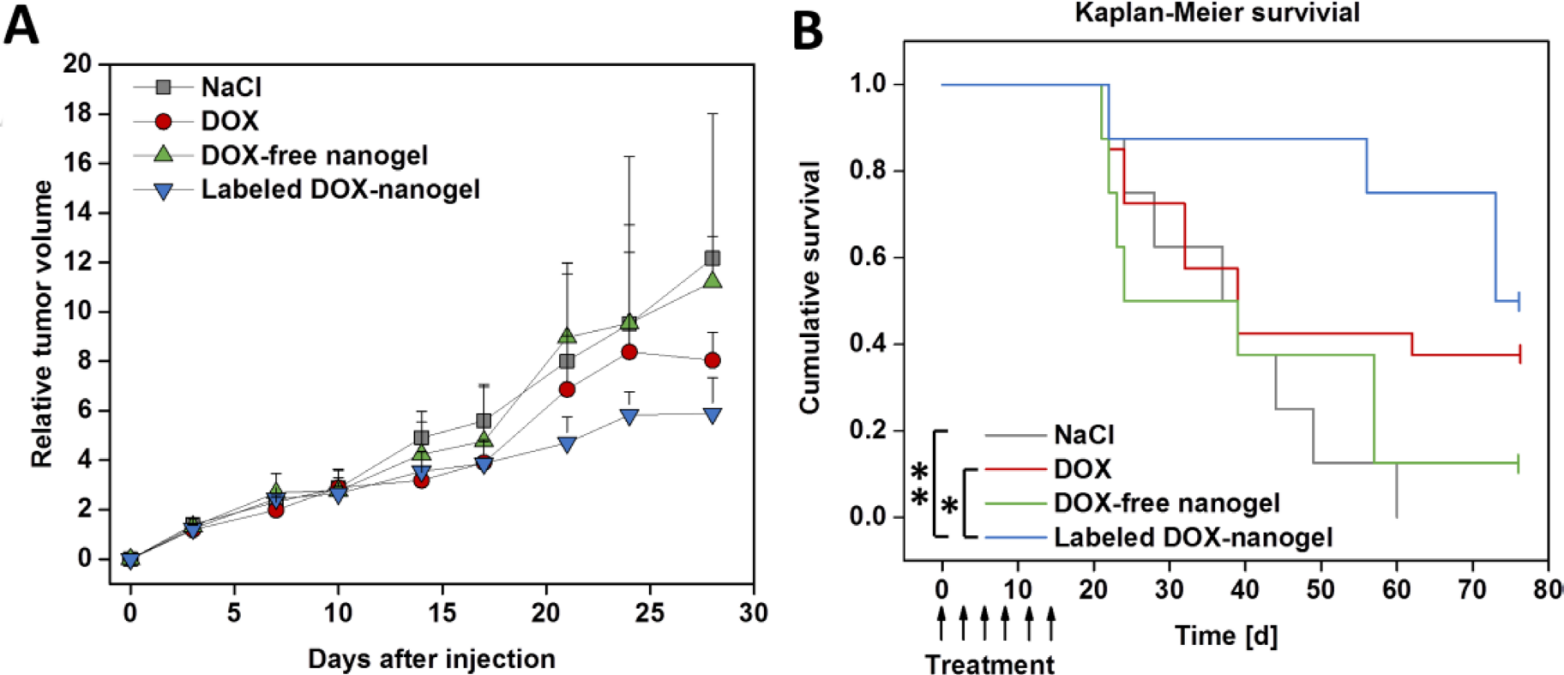
Anti-tumor activity of the DOX-nanogels was evaluated in a xenograft mouse model Male nude mice received a subcutaneous injection of HT-29 cells into the flank. When tumors reached 100 to 200 mm^3^ mice received 6 doses of 0.9 wt% NaCl, DOX (1 mg kg^–1^), DOX-free nanogel and labeled DOX-nanogel (corresponding to 1 mg kg^–1^ DOX) *via* tail vein injection from day 0 to day 15. (**A**) Development of the relative tumor volume is illustrated over time. Results are indicated as median + semi interquartile range. (**B**) Survival of mice bearing HT-29 derived tumors presented as a Kaplan–Meier survival curve. The individual endpoint of each animal was achieved when the tumor volume reached 1500 mm^3^. Statistical differences are displayed as ^*^p < 0.05 and ^**^p < 0.01 according to the log-rank test.

To test the therapeutic efficiency of labeled DOX-nanogels, a xenograft mouse model was established by subcutaneous injection of HT-29 cells. When the tumor volume reached 100–200 mm^3^ mice received 6 doses of drug or control every three days (day 0–15) according to a metronomic schedule. Mice were treated with saline (control), low dose of free DOX (1 mg kg^–1^), DOX-free nanogels and labeled DOX-nanogels (corresponding to 1 mg kg^–1^ DOX.). The absolute tumor volume was monitored until it reached the termination condition of 1500 mm^3^ (Supplementary Figure 18). No negative influence on the development of body weight was detected (Supplementary Figure 19). The individual time course of tumor development for each animal in the different treatment groups (*n* = 7–8) is shown in Figure 6A. The use of labeled DOX-nanogels reduced the tumor growth of mice compared to a treatment with NaCl, DOX-free nanogels or free DOX. These results are supported by the Kaplan–Meier survival of the HT-29 xenograft model (Figure 6B). Treatment with NaCl or DOX-free nanogels did not slow down the tumor growth, while the median survival time was 37 days for NaCl or 24 days for DOX-free nanogels, respectively. Administration of 1 mg kg^1^ DOX also had no effect on tumor inhibition compared to control groups with a median survival time of 39 days (*p* = 0.202). This might be attributed to the low DOX concentration used in this study. However, even though pure DOX did not seem to be able to reduce tumor progression in the xenograft model, the labeled DOX-nanogels were highly effective. Mice treated with labeled DOX-nanogels had a significant prolonged median survival time of 73 days compared to the NaCl control (*p* = 0.002) or pure DOX (*p* = 0.031). This might be explained by the more direct impact of DOX-nanogels on tumor tissue due to the EPR effect. As DOX is shielded within the nanogel, protected by a P(EtOx) shell, a prolonged circulation time can be expected, as shown for linear P(EtOx) [27]. These findings are in agreement with a recently published study by O. Sedlacek *et al.*, prolonging the median survival time of DOX-POx conjugates from 19 to 36 days [39]. However, the utilized DOX dose within the mentioned study was 20 mg kg^1^, while our nanogels already possess an effect at an administration of 1 mg kg^1^. With an equal or higher toxicity after cellular uptake, as demonstrated by *in vitro* investigations the nanogels are able to interfere with tumor growth more efficiently than the free drug. Combined with their excellent biocompatibility the presented drug carriers proved to be a promising material for cancer therapy.

## MATERIALS AND METHODS

### Material and instrumentation

Chemicals and solvents were purchased from Sigma-Aldrich, Merck, Fluka, and Acros. Hoechst 33342 trihydrochloride as well as LysoTracker^®^ Green DND-26 were obtained from Life Technologies (Thermo Fisher, Germany). 2-Ethyl-2-oxazoline (EtOx) and methyl tosylate (MeOTos) were distilled to dryness prior to use. EtOx was dried using barium oxide before distillation. 2-(4-((*tert*-Butoxycarbonyl)amino)butyl)-2-oxazoline (BocOx) was synthesized as described in a previous publication [57]. Consumables for cell culture, like pipettes and cell culture plates (96 well) were obtained from Greiner Bio-one (Austria/ Germany). If not stated otherwise, cell culture media and supplements (L-Glutamin, antibiotics) were obtained from Biochrom (Merck Millipore, Germany).

The Initiator Sixty single-mode microwave synthesizer from Biotage, equipped with a non-invasive IR sensor (accuracy: 2%), was used for polymerizations under microwave irradiation. Microwave vials were heated overnight to 110°C and allowed to cool to room temperature under an argon atmosphere before use. All polymerizations were carried out under temperature control. Size-exclusion chromatography (SEC) measurements of the protected polymers were performed on a Shimadzu system equipped with a SCL-10A system controller, a LC-10AD pump, a RID-10A refractive index detector and a PSS SDV column with chloroform/triethylamine (NEt_3_)/*iso*-propanol (94:4:2) as eluent. The column oven was set to 50°C. SEC of the deprotected statistical copolymers was performed on a Shimadzu system with a LC-10AD pump, a RID-10A refractive index detector, a system controller SCL-10A, a degasser DGU-14A, and a CTO-10A column oven using *N,N*-dimethyl acetamide (DMAc) with 2.1 g L^–1^ LiCl as the eluent and the column oven set to 50°C. Poly(styrene) (PS) samples were used as calibration standards for both solvent systems. Proton NMR spectroscopy (^1^H NMR) measurements were performed at room temperature on a Bruker AC 300 and 400 MHz spectrometer, using CDCl_3_ or *N,N-*dimethyl formamide (DMF)-D_7_ as solvents. Diffusion-ordered spectroscopy (DOSY) NMR measurements were performed at room temperature on a Bruker AC 400 MHz spectrometer using D_2_O as the deuterated solvent. The chemical shifts are given in ppm relative to the signal of the residual non-deuterated solvent.

Batch dynamic light scattering (DLS) was performed on a Zetasizer Nano ZS (Malvern Instruments, Herrenberg, Germany). All measurements were performed in folded capillary cells (DTS1071, Malvern Instruments, Herrenberg, Germany). After an equilibration time of 180 s, 3 × 30 s runs were carried out at 4°C, 25°C or 37°C (λ = 633 nm). If not stated explicitly, 25°C was used for measurements. The counts were detected at an angle of 173°. Each measurement was performed in triplicate. Apparent hydrodynamic radii, R_h_, were calculated according to the Stokes–Einstein equation.

Laser Doppler velocimetry was used to measure the electrokinetic potential, also known as zeta potential. The measurements were performed on a Zetasizer Nano ZS (Malvern Instruments, Herrenberg, Germany) in folded capillary cells (DTS1071). For each measurement, 15 runs were carried out using the fast-field and slow-field reversal mode at 150 V. Each experiment was performed in triplicate at 25°C. The zeta potential (ζ) was calculated from the electrophoretic mobility (μ) according to the Henry Equation [58]. The Henry coefficient, f(ka), was calculated according to Ohshima [59].

cryoTEM investigations were conducted with a FEI Tecnai G^2^ 20 at 200 kV acceleration voltage. Specisms were vitrified by a Vitrobot Mark V system on Quantifoil grids (R2/2). The blotting time was 1 s with blotting force offset of 0. The amount of solution was 7 μL. Samples were plunge frozen in liquid ethane and stored under liquid nitrogen until transferred to the Gatan cryo-holder and brought into the microscope. Images were acquired with a 4k × 4k CCD Eagle camera.

Absorbance and fluorescence spectra were recorded using a Tecan Infinite M200 Pro micro plate reader (Crailsheim, Germany) by the use of black well plates with a flat and transparent bottom.

### Block copolymer of 2-ethyl-2-oxazoline (EtOx) and 2-(4-((tert-butoxycarbonyl)amino)butyl)-2-oxazoline (BocOx) (P(EtOx-*b*-BocOx)), (1)

In a microwave vial, EtOx (757 μL, 7.5 mmol), MeTos (16.2 μL, 0.107 mmol) and acetonitrile (3.4 mL) were mixed under inert conditions. After heating in the microwave synthesizer at 140°C for 25 min the vial was introduced into a glove box with nitrogen atmosphere, a sample was taken for NMR and SEC measurements and BocOx (803 μL, 3.2 mmol) was added. The closed vial was heated again in the microwave synthesizer (140°C, 20 min). The solution was precipitated in cold (−80°C, 300 mL) diethyl ether. The white precipitate was filtered and dried in high vacuum (1.4 g, 92%).

^1^H NMR (CDCl_3_, 300 MHz) (6): δ = 7.66, (d, 8.1 Hz, 0.019 H, tosylate), 7.14 (d, 8.21 Hz, 0.019 H, tosylate), 3.45 (s, 4 H, backbone), 3.10 (s, 0.58 H, CH_2_-CH_2_-NH (BocOx)), 2.50–2.15 (m, 1.96 H, CH_2_ (EtOx)/CH_2_-CH_2_-NHBoc), 1.62 (s, 0.52 H, CH_2_-CH_2_-CH_2_ (BocOx)), 1.52 (s, 0.52 H, CH_2_-CH_2_-CH_2_ (BocOx)), 1.42 (s, 2.3 H, CH_3_ (BocOx)), 1.21 (s, 2.1 H, CH_3_ (EtOx)) ppm.

SEC (eluent: CHCl_3_/*iso*-propanol/NEt_3_, PS-standard): M_n_ = 8,200 g mol^–1^, M_w_ = 9.900 g mol^–1^, Ð = 1.07.

### Deprotection of (P(EtOx-*b*-BocOx)) (1) to yield (P(EtOx-*b*-AmOx)), (2)

P(EtOx-*b*-BocOx) (1, 1.3 g) was dissolved in TFA (5 mL) and heated to 60°C for 1 h. After stirring for 12 h at room temperature, the mixture was diluted with 10 mL methanol and precipitated in 400 mL of cold (−80°C) diethyl ether. The precipitate was re-dissolved in methanol (100 mL) and stirred with Amberlyst A21 for 48 h. Subsequently, the solvent was removed, the polymer was dissolved in de-ionized water and freeze dried (−80°C, 0.003 mbar). The polymer was obtained as white powder (1.2 g, 92%).

^1^H NMR (DMF-D_7_, 300 MHz) (2): δ = 4.9 (s, 2.3 H, NH_2_), 3.51 (s, 4 H, backbone), 3.07 (s, 0.49 H, CH_2_-CH_2_-NH_2_), 2.44 (m, 2.1 H, CH_2_ (EtOx)/CH_2_-CH_2_-CO (AmOx)), 1.9–1.54 (m, 0.96 H, CH_2_-CH_2_-CH_2_-CH_2_ (AmOx)), 1.2 (s, 2,3 H, CH_3_ (EtOx)) ppm.

SEC (eluent: DMAc/LiCl, PS-standard): M_n_ = 13,900 g mol^–1^, Ð = 1.11.

### Labeling of (P(EtOx-*b*-AmOx)) (2) using Alexafluor 660, (3)

P(EtOx-*b*-AmOx) (2, 14 mg) was dissolved in DMF (5 mL) and Alexfluor 660^®^ (1 mg, ~1 eq. per macromolecule) as well as triethyl amine (1 μL) were added under stirring. The solution was stirred at room temperature overnight and subsequently precipitated in cold diethyl ether, (300 mL, –80°C). The precipitated was filtered off, dissolved in water and transferred to a dialysis tube (6,000 to 8,000 g mol^–1^ cut off, Spectra/Por^®^). The polymer was dialysed against water until the solution outside the tube stayed colorless. After freeze drying, the product was obtained as deep blue powder (8 mg, 53%, degree of functionalization = 30%).

SEC (eluent: DMAc/LiCl, PS-standard): M_n_ = 14,600 g mol^–1^, Ð = 1.11.

UV/Vis: λ_Abs_ = 660 nm, λ_Em_ (excitation at 600 nm) = 690 nm.

### Self-assembly and cross-linking

To create nanostructures, the unlabeled block copolymer (2, 90 mg, 0.006 mmol) or a mixture of the polymers 2 and 3 (9:1, 90 mg, 0.006 mmol) were dissolved in CHCl_3_, (5 mg mL^–1^) and stirred for 3 h. Subsequently, glutaraldehyde (30 mg, 0.3 mmol, 1.5 eq. per amine) was added and the solution was stirred another 3 h. With proceeding reaction time the colour of the solution changed from colourless to yellow. To quench the excess of aldehyde functionalities, 6-amino fluorescein (50 mg) or DOX (50 mg) were added, respectively, and stirred for 12 h. Subsequently, the amount of solvent was reduced under an argon stream and the residual was precipitated in 100 mL cold diethyl ether (−80°C). To purify the self-assembled structures from residual capping agent and cross-linker, dialysis in MeOH/water (1:4) was applied using a membrane with a molar mass cut off of 3,500 g mol^–1^ (Roth Zellutrans). After the extraction was finished, the dialysis medium was changed to pure water and the aqueous solution was freeze dried to yield an orange or, in the case of DOX, a red powder.

### Determination of dye loading content by absorbance/fluorescence

The absorbance/fluorescence of 6AF loaded nanostructures was investigated under alkaline conditions (1 mol L^–1^ NaOH in water) in diluted solution (0.1 mg mL^–1^). The absorbance was determined at a wavelength of 490 nm and compared to a dilution series of 6AF in the same aqueous NaOH solution. To the 6AF stock solution a 100 fold excess of glutaraldehyde was added to ensure that only the imine species of 6AF is present. Emission was detected at an excitation wavelength of λ = 450 nm. Micellar samples as well as 6AF calibration exhibit an emission maximum at λ = 510 nm.

DOX conjugated samples were measured in water (0.1 mg mL^–1^) and compared to a dilution series of DOX in water. All measurements were carried out in a 96 well-plate format with 200 μL per well and double determination for each measuring point. The read out was accomplished using a Tecan Infinite M200 Pro micro plate reader (Crailsheim, Germany).

### Determination of the nanogel stability

Labeled DOX-nanogels were dissolved in 150 mM phosphate buffered saline (PBS) buffer (pH = 7.4) and measured by means of size (z-average and number mean) and uniformity (PDI) using DLS measurements as described above. Measurements were conducted at 4°C or 37°C, and nanogel solutions were stored at the respective temperature in between measurements.

### Determination of the DOX release

Labeled DOX-nanogels were dissolved in 0.9 wt % NaCl or 150 mM phosphate buffered saline (PBS) buffer (pH = 5.0) containing 200 mM glycine. Qualitative DOX release was determined using DOSY NMR measurements as described above. A sample containing pure DOX dissolved in 0.9 wt % NaCl was used for comparison.

### Determination of the cytotoxicity by XTT assay

Cytotoxicity studies were performed with the sensitive mouse fibroblast cell line L929, as recommended by ISO10993-5, and with the human colorectal adenocarcinoma cell line HT-29. The L929 cells were routinely cultured in Dulbecco's modified eagle's medium (DMEM) and HT-29 cells in RPMI 1640 supplemented with 10% fetal calf serum (FCS), 100 U mL^–1^ penicillin and 100 μg mL^–1^ streptomycin at 37°C in a humidified 5% (v/v) CO_2_ atmosphere. Cells were seeded at 10^4^ cells per well in a 96-well plate and incubated for 24 h, whereas no cells were seeded in the outer wells. Afterwards, the testing substances (nanogels or DOX) at indicated end concentrations were added to the cells and the plates were incubated for further 24 h. Subsequently, a XTT assay (Cell Proliferation Kit II, Roche Diagnostics) was performed according to supplier's information. After a further incubation of 4 h, the absorbance was measured at a wavelength of λ = 450 nm and a reference wavelength of λ = 630 nm with untreated cells on the same well plate serving as negative controls. The negative control was standardized as 0% of metabolism inhibition and referred as 100% viability. Cell viability below 70% was considered indicative of cytotoxicity. Data are expressed as mean ± SD of six determinations. The half maximal inhibitory concentration (IC_50_) was calculated with the GraphPad Prism Software.

### Blood compatibility measurements

To assess the hemolytic activity of the polymer solutions, blood from sheep, collected in heparinized-tubes (Institut für Versuchstierkunde und Tierschutz/Laboratory of Animal Science and Animal Welfare, Friedrich Schiller University Jena), was centrifuged at 4500 × g for 5 min, and the pellet was washed three times with cold 1.5 mmol L^–1^ phosphate buffered saline (PBS, pH 7.4). After dilution with PBS in a ratio of 1:7, aliquots of erythrocyte suspension were mixed 1:1 with the polymer solution and incubated in a water bath at 37°C for 60 min. After centrifugation at 2400 × g for 5 min the hemoglobin release into the supernatant was determined spectrophotometrically using a microplate reader (TECAN Infinite M200 PRO) at λ = 544 nm wavelength. Complete hemolysis (100%) was achieved using 1% Triton X-100 serving as positive control. Thereby, PBS served as negative control (0%). A value less than 2% hemolysis rate was taken as non-hemolytic. Experiments were run in triplicates and were performed with three different blood donors.

For the examination of the erythrocyte aggregation, erythrocytes were isolated as described above. An erythrocytes suspension was mixed with the same volume of polymer solution in a clear flat bottomed 96-well plate. The cells were incubated at 37°C for 2 h, and the absorbance was measured at λ = 645 nm in a microplate reader (TECAN Infinite M200 Pro). 25 kDa *b*PEI (50 μg mL^–1^) was used as positive control and PBS treated cells served as negative control. Absorbance values of the test solutions lower than negative control were regarded as aggregation. Experiments are the result of triplicates and were performed with three different donor blood batches.

### Confocal microscopy

For live CLSM analysis of cell uptake, HT-29 cells (0.2 × 10^6^ cells mL^–1^) were seeded in glass-bottomed, 4-chamber dishes (CELLVIEW, Greiner Bio-One) and cultured for 24 h. One hour prior to nanogel/ drug treatment, a media change with fresh culture media occurred. Cells were incubated with nanogel or DOX (10 μg mL^–1^) for 6 h or 24 h, respectively. For examination of nanogel/ drug co-localization with cell organelles, the lysosomes were stained with LysoTracker Green^®^ DND-26 and the cell nuclei were counterstained with Hoechst 33342. Live cell CLSM images were acquired using a Zeiss LSM 880, Elyra PS.1 system (Carl Zeiss, Germany) with excitation wavelengths/emission filters of 405nm/BP 405–480 nm for Hoechst 33342, 488 nm/BP 505 to 530 nm for LysoTracker^®^ Green DND-26 and 488 nm/BP 585 to 615 nm for DOX and 633 nm/BP 724 to 777 nm for Alexafluor 660^®^. Images were captured with a 1.4 NA Plan-Apochromat 63 × oil objective and in multitrack mode, enabling single excitation and emission of fluorescence dyes. Co-localization was visualized in overlay images of the multiple channels.

The imaging of histological tissue sections (heart, liver, kidney, tumor) were performed with excitation wavelengths/ emission filters of 488 nm/BP 580 to 615 nm and a 1.4 NA Plan-Apochromat 40 × oil objective.

### Cellular uptake studies

The evaluation of the nanogel and free DOX uptake was performed by flow cytometry (FC) measured on a Beckmann Coulter Cytomics FC-500 equipped with an Uniphase Argon ion laser (488 nm, 20 mW output) and analyzed with the Cytomics CXP software. In brief, HT-29 cells (0.2 × 10^6^ cells mL^–1^ seeded in 24-well plates) were incubated for 6 h and 24 h with labeled labeled DOX-nanogel or free DOX (0.01 mg mL^–1^) at 37°C or 4°C, respectively. In the case of the 4°C uptake study, cell culture media was supplemented with 15 mM HEPES (4-(2-hydroxyethyl)-1-piperazineethanesulfonic acid, Biochrom, Merck) as buffering agent. Afterwards, cells were harvested by trypsinization and trypan blue (1:10) was added to quench the outer fluorescence. 10^4^ cells were measured by flow cytometry, whereby the number of all viable cells, showing signals at 575 nm, were gated. Cells incubated with culture medium only served as control. The experiments were performed at least three times independently.

### Animals

Male athymic nude mice (Crl:CD1-Foxn1^nu^), 6 to 8 weeks age, were purchased by Charles River and were kept in a standard pathogen-free barrier facility accredited by the Association for Assessment and Accreditation of Laboratory Animal Care. All experiments were approved by the local Institutional Animal Care and Use Committee (Jena, 02-011/15). Mice had free access to standard chow and tap water at all times. Body weight and tumor size (measured with a digital caliper) were monitored twice a week. Tumor volume was calculated with the formula (L × W^2^)/2, were L is the longest and W the shortest diameter (mm) of the tumor.

### *In vivo* toxicity and biodistribution

Safety evaluation of the nanogels was carried out on healthy male nude mice without tumors, which were randomly assigned to 3 groups (4 mice per group). A single dose (150 μl) of saline (control) or nanogels corresponding to a DOX concentration of 0.3 and 1 mg kg^–1^ body weight were injected *via* tail vein. Body weight, animal constitution and physical activity were monitored for 2 weeks.

For biodistribution experiments HT-29 cells (1 × 10^6^ in 250 μl) were injected subcutaneously into the flank of nude mice. Mice bearing tumors approximately 6–8 mm received a single dose (150 μl) of saline or nanogels with a DOX concentration of 1 mg kg^–1^ *via* tail vein injection. At 6, 48 and 72 h after injection mice were sacrificed and tumor, heart, liver and kidney were excised for further analysis, immediately frozen with liquid nitrogen and stored at –70°C prior to tissue sectioning. Single tissue sections (8 μm thickness) of organs and tumors were cut with a CM 1860 Crystat (Leica Biosystems, Wetzlar, Germany), air-dried on glass slides and embedded in a water-based mounting media (Aquatex, Merck).

### Anti-tumor activity *in vivo*

The xenograft model was established by subcutaneous injection of HT-29 cells (1 × 10^6^ in 250 μl) into the flank of male nude mice. When tumors reached a volume of 100–200 mm^3^ mice were assigned to 4 treatment groups (10 mice per group) with no significant differences in body weight or tumor volume between the groups. Mice were injected with treatment solutions (saline, 1 mg kg^–1^ DOX, labeled DOX loaded nanogel (6) (corresponding to 1 mg kg^–1^ DOX), and Dox-free nanogel at the same concentration as nanogel 6) *via* tail vein injection on day 0, 3, 6, 9, 11 and 15. Mice were sacrificed when the tumor volume reached 1500 mm^3^, which was determined as the individual end point of the survival curve. After sacrifice tumors were excised and weighed. Mice reaching any termination condition (maximum tumor volume, weight loss over 15%, infected wound or limited mobility) before the end of the treatment period were excluded from the survival study.

### Statistical analysis

The values represent the mean ± SD (standard deviation). For uptake studies direct comparison of two different groups was done with two-tailed, non-paired Student's *t*-test. A value of *p* < 0.05 was considered as statistically significant. The body weight or tumor volume of the nude mice were tested regarding normal distribution and homogeneity of variances with the IBM SPSS software. Statistical differences were calculated according to a one-way ANOVA. Survival analysis was performed with SPSS and calculated with the Kaplan–Meier method. Significant differences were assessed with the log-rank test. A value of *p* < 0.05 was considered as statistically significant.

## CONCLUSIONS

Within this report, a straightforward approach to POx based nanogels, covalently loaded with the anti-cancer drug DOX is presented. Nanogels were synthesized *via* cross-linking of a block copolymer micelle with a cationic poly(2-(4-aminobutyl)-2-oxazoline) core and a poly(2-ethyl-2-oxazoline) shell. Cross-linking as well as drug loading was accomplished by pH responsive imine chemistry. Moreover, the amine groups of the drug delivery system allowed the irreversible labeling with a near infra-red fluorescent dye. In *in vitro* studies DOX loaded POx based nanogels showed a toxicity profile comparable to the free drug, while unloaded drug carriers showed no toxicity. The blood compatibility of the drug delivery system was found to be suitable for the envisioned application, therefore the cellular uptake was investigated by flow cytometry and fluorescence microscopy. While the amount of internalized drug was enhanced when incorporated into a nanogel, the release of the drug into the nucleus was delayed compared to free DOX. This is beneficial as a lower amount of drug is required to yield the same effect. Furthermore, the nanogels were shown to be more tumor specific than DOX, which reduces side-effects during therapy. *In vivo* investigation on xenograft mouse models were conducted to assess the ability of the designed system to reduce tumor growth. In combination to the new nanogel-based drug delivery system a metronomic schedule of DOX treatment was applied. Initial studies on healthy mice showed no adverse effects of the DOX-free nanogels or low dosed labeled DOX-nanogels on body weight and behavior. The biodistribution was investigated by microscopy of organs of mice treated with labeled DOX-nanogels and showed a localization of DOX within tumorous tissue, most likely associated to the enhanced permeability and retention (EPR) effect. Finally, the therapeutic efficiency of the POx based drug delivery system was investigated in a survival study of xenograft mice. While the low doses of pure DOX did not show a significant reduction in tumor progression, the metronomic schedule of the labeled DOX-nanogels proved a significant tumor growth inhibition and increase in survival time. Future studies will focus on detailed investigations of the pharmacokinetics of the presented system as well as on studying the biocompatibility of higher drug doses.

## SUPPLEMENTARY MATERIALS TABLE AND FIGURES



## References

[1] Gewirtz DA (1999). A critical evaluation of the mechanisms of action proposed for the antitumor effects of the anthracycline antibiotics adriamycin and daunorubicin. Biochem Pharmacol.

[2] Thorn CF, Oshiro C, Marsh S, Hernandez-Boussard T, McLeod H, Klein TE, Altman RB (2011). Doxorubicin pathways: pharmacodynamics and adverse effects. Pharmacogenet Genomics.

[3] Yang F, Teves SS, Kemp CJ, Henikoff S (2014). Doxorubicin, DNA torsion, and chromatin dynamics. Biochim Biophys Acta.

[4] Lipshultz SE, Scully RE, Lipsitz SR, Sallan SE, Silverman LB, Miller TL, Barry EV, Asselin BL, Athale U, Clavell LA, Larsen E, Moghrabi A, Samson Y (2010). Assessment of dexrazoxane as a cardioprotectant in doxorubicin-treated children with high-risk acute lymphoblastic leukaemia: long-term follow-up of a prospective, randomised, multicentre trial. Lancet Oncol.

[5] He Z, Wan X, Schulz A, Bludau H, Dobrovolskaia MA, Stern ST, Montgomery SA, Yuan H, Li Z, Alakhova D, Sokolsky M, Darr DB, Perou CM (2016). A high capacity polymeric micelle of paclitaxel: implication of high dose drug therapy to safety and in vivo anti-cancer activity. Biomaterials.

[6] Luxenhofer R, Han Y, Schulz A, Tong J, He Z, Kabanov AV, Jordan R (2012). Poly(2-oxazoline)s as polymer therapeutics. Macromol Rapid Commun.

[7] Bertrand N, Wu J, Xu X, Kamaly N, Farokhzad OC (2014). Cancer nanotechnology: the impact of passive and active targeting in the era of modern cancer biology. Adv Drug Deliv Rev.

[8] Eckmann DM, Composto RJ, Tsourkas A, Muzykantov VR (2014). Nanogel carrier design for targeted drug delivery. J Mater Chem B Mater Biol Med.

[9] Pathak RK, Wen R, Kolishetti N, Dhar S (2017). A prodrug of two approved drugs, cisplatin and chlorambucil, for chemo war against cancer. Mol Cancer Ther.

[10] Wen R, Banik B, Pathak RK, Kumar A, Kolishetti N, Dhar S (2016). Nanotechnology inspired tools for mitochondrial dysfunction related diseases. Adv Drug Deliv Rev.

[11] Wen R, Dhar S (2016). Turn up the cellular power generator with vitamin E analogue formulation. Chem Sci (Camb).

[12] Sultana F, Imran-Ul-Haque M, Arafat M, Sharmin S (2013). An Overview of Nanogel Drug Delivery System. J Appl Pharm Sci.

[13] Kronek J, Kroneková Z, Lustoň J, Paulovičová E, Paulovičová L, Mendrek B (2011). *In vitro* bio-immunological and cytotoxicity studies of poly(2-oxazolines). J Mater Sci Mater Med.

[14] Eskow Jaunarajs KL, Standaert DG, Viegas TX, Bentley MD, Fang Z, Dizman B, Yoon K, Weimer R, Ravenscroft P, Johnston TH, Hill MP, Brotchie JM, Moreadith RW (2013). Rotigotine polyoxazoline conjugate SER-214 provides robust and sustained antiparkinsonian benefit. Mov Disord.

[15] Wilson P, Chun Ke P, Davis TP, Kempe K (2017). Poly(2-oxazoline)-based micro- and nanoparticles: A review. Eur Polym J.

[16] Chen F, Zhang J, Wang L, Wang Y, Chen M (2015). Tumor pH(e)-triggered charge-reversal and redox-responsive nanoparticles for docetaxel delivery in hepatocellular carcinoma treatment. Nanoscale.

[17] Dams ET, Laverman P, Oyen WJ, Storm G, Scherphof GL, van Der Meer JW, Corstens FH, Boerman OC (2000). Accelerated blood clearance and altered biodistribution of repeated injections of sterically stabilized liposomes. J Pharmacol Exp Ther.

[18] Chanan-Khan A, Szebeni J, Savay S, Liebes L, Rafique NM, Alving CR, Muggia FM (2003). Complement activation following first exposure to pegylated liposomal doxorubicin (Doxil): possible role in hypersensitivity reactions. Ann Oncol.

[19] Armstrong JK, Hempel G, Koling S, Chan LS, Fisher T, Meiselman HJ, Garratty G (2007). Antibody against poly(ethylene glycol) adversely affects PEG-asparaginase therapy in acute lymphoblastic leukemia patients. Cancer.

[20] Rudmann DG, Alston JT, Hanson JC, Heidel S (2013). High molecular weight polyethylene glycol cellular distribution and PEG-associated cytoplasmic vacuolation is molecular weight dependent and does not require conjugation to proteins. Toxicol Pathol.

[21] Bendele A, Seely J, Richey C, Sennello G, Shopp G (1998). Short communication: renal tubular vacuolation in animals treated with polyethylene-glycol-conjugated proteins. Toxicol Sci.

[22] Baumann A, Tuerck D, Prabhu S, Dickmann L, Sims J (2014). Pharmacokinetics, metabolism and distribution of PEGs and PEGylated proteins: quo vadis?. Drug Discov Today.

[23] Luxenhofer R, Sahay G, Schulz A, Alakhova D, Bronich TK, Jordan R, Kabanov AV (2011). Structure-property relationship in cytotoxicity and cell uptake of poly(2-oxazoline) amphiphiles. J Control Release.

[24] Kronek J, Paulovičová E, Paulovičová L, Kroneková Z, Lustoň J (2012). Immunomodulatory efficiency of poly(2-oxazolines). J Mater Sci Mater Med.

[25] Zalipsky S, Hansen CB, Oaks JM, Allen TM (1996). Evaluation of blood clearance rates and biodistribution of poly(2-oxazoline)-grafted liposomes. J Pharm Sci.

[26] Woodle MC, Engbers CM, Zalipsky S (1994). New amphipatic polymer-lipid conjugates forming long-circulating reticuloendothelial system-evading liposomes. Bioconjug Chem.

[27] Wyffels L, Verbrugghen T, Monnery BD, Glassner M, Stroobants S, Hoogenboom R, Staelens S (2016). μPET imaging of the pharmacokinetic behavior of medium and high molar mass (89)Zr-labeled poly(2-ethyl-2-oxazoline) in comparison to poly(ethylene glycol). J Control Release.

[28] Glassner M, Palmieri L, Monnery BD, Verbrugghen T, Deleye S, Stroobants S, Staelens S, Wyffels L, Hoogenboom R (2017). The Label Matters: μPET Imaging of the Biodistribution of Low Molar Mass 89Zr and 18F-Labeled Poly(2-ethyl-2-oxazoline). Biomacromolecules.

[29] Guillerm B, Monge S, Lapinte V, Robin JJ (2012). How to modulate the chemical structure of polyoxazolines by appropriate functionalization. Macromol Rapid Commun.

[30] Hartlieb M, Kempe K, Schubert US (2015). Covalently cross-linked poly(2-oxazoline) materials for biomedical applications - from hydrogels to self-assembled and templated structures. J Mater Chem B Mater Biol Med.

[31] Hartlieb M, Pretzel D, Wagner M, Hoeppener S, Bellstedt P, Görlach M, Englert C, Kempe K, Schubert US (2015). Core cross-linked nanogels based on the self-assembly of double hydrophilic poly(2-oxazoline) block copolymers. J Mater Chem B Mater Biol Med.

[32] Hartlieb M, Bus T, Kübel J, Pretzel D, Hoeppener S, Leiske MN, Kempe K, Dietzek B, Schubert US (2017). Tailoring Cellular Uptake and Fluorescence of Poly(2-oxazoline)-Based Nanogels. Bioconjug Chem.

[33] Xin Y, Yuan J (2012). Schiff's base as a stimuli-responsive linker in polymer chemistry. Polym Chem.

[34] Kareva I, Waxman DJ, Lakka Klement G (2015). Metronomic chemotherapy: an attractive alternative to maximum tolerated dose therapy that can activate anti-tumor immunity and minimize therapeutic resistance. Cancer Lett.

[35] Romiti A, Cox MC, Sarcina I, Di Rocco R, D’Antonio C, Barucca V, Marchetti P (2013). Metronomic chemotherapy for cancer treatment: a decade of clinical studies. Cancer Chemother Pharmacol.

[36] Fiallo M, Laigle A, Borrel MN, Garnier-Suillerot A (1993). Accumulation of degradation products of doxorubicin and pirarubicin formed in cell culture medium within sensitive and resistant cells. Biochem Pharmacol.

[37] Mohan P, Rapoport N (2010). Doxorubicin as a molecular nanotheranostic agent: effect of doxorubicin encapsulation in micelles or nanoemulsions on the ultrasound-mediated intracellular delivery and nuclear trafficking. Mol Pharm.

[38] Meyer CD, Joiner CS, Stoddart JF (2007). Template-directed synthesis employing reversible imine bond formation. Chem Soc Rev.

[39] Sedlacek O, Monnery BD, Mattova J, Kucka J, Panek J, Janouskova O, Hocherl A, Verbraeken B, Vergaelen M, Zadinova M, Hoogenboom R, Hruby M (2017). Poly(2-ethyl-2-oxazoline) conjugates with doxorubicin for cancer therapy: in vitro and in vivo evaluation and direct comparison to poly[N-(2-hydroxypropyl)methacrylamide] analogues. Biomaterials.

[40] Basuki JS, Duong HT, Macmillan A, Erlich RB, Esser L, Akerfeldt MC, Whan RM, Kavallaris M, Boyer C, Davis TP (2013). Using fluorescence lifetime imaging microscopy to monitor theranostic nanoparticle uptake and intracellular doxorubicin release. ACS Nano.

[41] Amelio I, Cutruzzolá F, Antonov A, Agostini M, Melino G (2014). Serine and glycine metabolism in cancer. Trends Biochem Sci.

[42] Leiske MN, Sobotta FH, Richter F, Hoeppener S, Brendel JC, Traeger A, Schubert US (2018). How to tune the gene delivery and biocompatibility of poly(2-(4-aminobutyl)-2-oxazoline) by self and co assembly. Biomacromolecules.

[43] Thonemann B, Schmalz G, Hiller KA, Schweikl H (2002). Responses of L929 mouse fibroblasts, primary and immortalized bovine dental papilla-derived cell lines to dental resin components. Dent Mater.

[44] Misra R, Sahoo SK (2010). Intracellular trafficking of nuclear localization signal conjugated nanoparticles for cancer therapy. Eur J Pharm Sci.

[45] Cai S, Alhowyan AA, Yang Q, Forrest WC, Shnayder Y, Forrest ML (2014). Cellular uptake and internalization of hyaluronan-based doxorubicin and cisplatin conjugates. J Drug Target.

[46] Yildirim T, Traeger A, Sungur P, Hoeppener S, Kellner C, Yildirim I, Pretzel D, Schubert S, Schubert US (2017). Polymersomes with Endosomal pH-Induced Vesicle-to-Micelle Morphology Transition and a Potential Application for Controlled Doxorubicin Delivery. Biomacromolecules.

[47] Kubota T, Furukawa T, Tanino H, Suto A, Otan Y, Watanabe M, Ikeda T, Kitajima M (2001). Resistant mechanisms of anthracyclines—pirarubicin might partly break through the P-glycoprotein-mediated drug-resistance of human breast cancer tissues. Breast Cancer.

[48] Mi Y, Lou L (2007). ZD6474 reverses multidrug resistance by directly inhibiting the function of P-glycoprotein. Br J Cancer.

[49] Upadhyay KK, Bhatt AN, Mishra AK, Dwarakanath BS, Jain S, Schatz C, Le Meins JF, Farooque A, Chandraiah G, Jain AK, Misra A, Lecommandoux S (2010). The intracellular drug delivery and anti tumor activity of doxorubicin loaded poly(γ-benzyl L-glutamate)-b-hyaluronan polymersomes. Biomaterials.

[50] Upadhyay KK, Le Meins JF, Misra A, Voisin P, Bouchaud V, Ibarboure E, Schatz C, Lecommandoux S (2009). Biomimetic doxorubicin loaded polymersomes from hyaluronan-block-poly(γ-benzyl glutamate) copolymers. Biomacromolecules.

[51] Yildirim T, Traeger A, Preussger E, Stumpf S, Fritzsche C, Hoeppener S, Schubert S, Schubert US (2016). Dual Responsive Nanoparticles from a RAFT Copolymer Library for the Controlled Delivery of Doxorubicin. Macromolecules.

[52] Hyung Park J, Kwon S, Lee M, Chung H, Kim JH, Kim YS, Park RW, Kim IS, Bong Seo S, Kwon IC, Young Jeong S (2006). Self-assembled nanoparticles based on glycol chitosan bearing hydrophobic moieties as carriers for doxorubicin: in vivo biodistribution and anti-tumor activity. Biomaterials.

[53] Gao Y, Li Y, Li Y, Yuan L, Zhou Y, Li J, Zhao L, Zhang C, Li X, Liu Y (2015). PSMA-mediated endosome escape-accelerating polymeric micelles for targeted therapy of prostate cancer and the real time tracing of their intracellular trafficking. Nanoscale.

[54] Qiu LY, Yan L, Zhang L, Jin YM, Zhao QH (2013). Folate-modified poly(2-ethyl-2-oxazoline) as hydrophilic corona in polymeric micelles for enhanced intracellular doxorubicin delivery. Int J Pharm.

[55] Seymour LW, Ulbrich K, Strohalm J, Kopeček J, Duncan R (1990). The pharmacokinetics of polymer-bound adriamycin. Biochem Pharmacol.

[56] Wang J, Masehi-Lano JJ, Chung EJ (2017). Peptide and antibody ligands for renal targeting: nanomedicine strategies for kidney disease. Biomater Sci.

[57] Hartlieb M, Pretzel D, Kempe K, Fritzsche C, Paulus RM, Gottschaldt M, Schubert US (2013). Cationic poly(2-oxazoline) hydrogels for reversible DNA binding. Soft Matter.

[58] Delgado AV, González-Caballero F, Hunter RJ, Koopal LK, Lyklema J, International Union of Pure and Applied Chemistry, Physical and Biophysical Chemistry Division IUPAC Technical Report (2007). Measurement and interpretation of electrokinetic phenomena. J Colloid Interface Sci.

[59] Ohshima H (1994). A Simple Expression for Henry's Function for the Retardation Effect in Electrophoresis of Spherical Colloidal Particles. J Colloid Interface Sci.

