# Supplementary material for: Tumor targeting with pH-responsive poly(2-oxazoline)-based nanogels for metronomic doxorubicin treatment
Source: Oncotarget. 2018 Apr 27;9(32):22316–31. doi: 10.18632/oncotarget.24806 (PMC5976466; doi:10.18632/oncotarget.24806)
Supplement: Supplementary file 1 [file oncotarget-09-22316-s001.pdf]

Tumor targeting with pH-responsive poly(2-oxazoline)-based nanogels for metronomic doxorubicin treatment

SUPPLEMENTARY MATERIALS

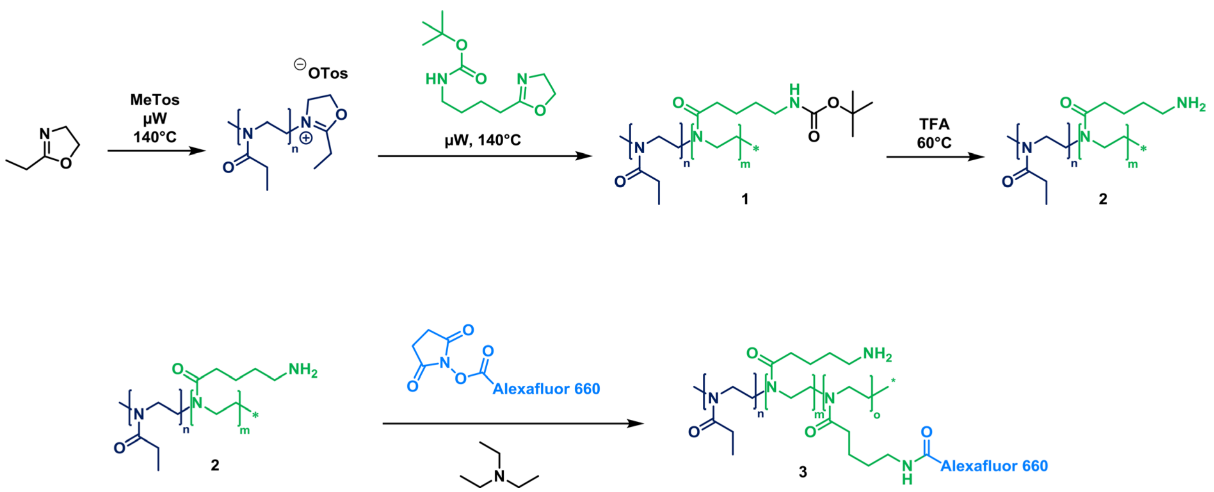

Supplementary Scheme 1: Schematic representation of the production of poly(2-oxazoline) block copolymers using cationic ring opening polymerization followed by deprotection of the Boc protected amine groups. Labelling was conducted by amidation of amine groups with Alexafluor 660 NHS ester.

Supplementary Table 1: Composition and analytical data of the POx block copolymers

Sample	NMR		SEC	
	Composition	M_n [g mol^{-1}]	M_n [g mol^{-1}]	Đ
1[a]	P(EtOx$_{98}$-*b*-BocOx$_{32}$)	17,500	8,200	1.07
2[b]	P(EtOx$_{98}$-*b*-AmOx$_{32}$)	14,200	13,900	1.11
3[b]	P(EtOx$_{98}$-*b*-[AmOx31-*stat*-FOx$_{1}$])	15,300	14,100	1.12

[a]SEC measurement in $CHCl_3$; [b]SEC Measurement in DMAc. A poly(styrene) calibration was used in both cases.

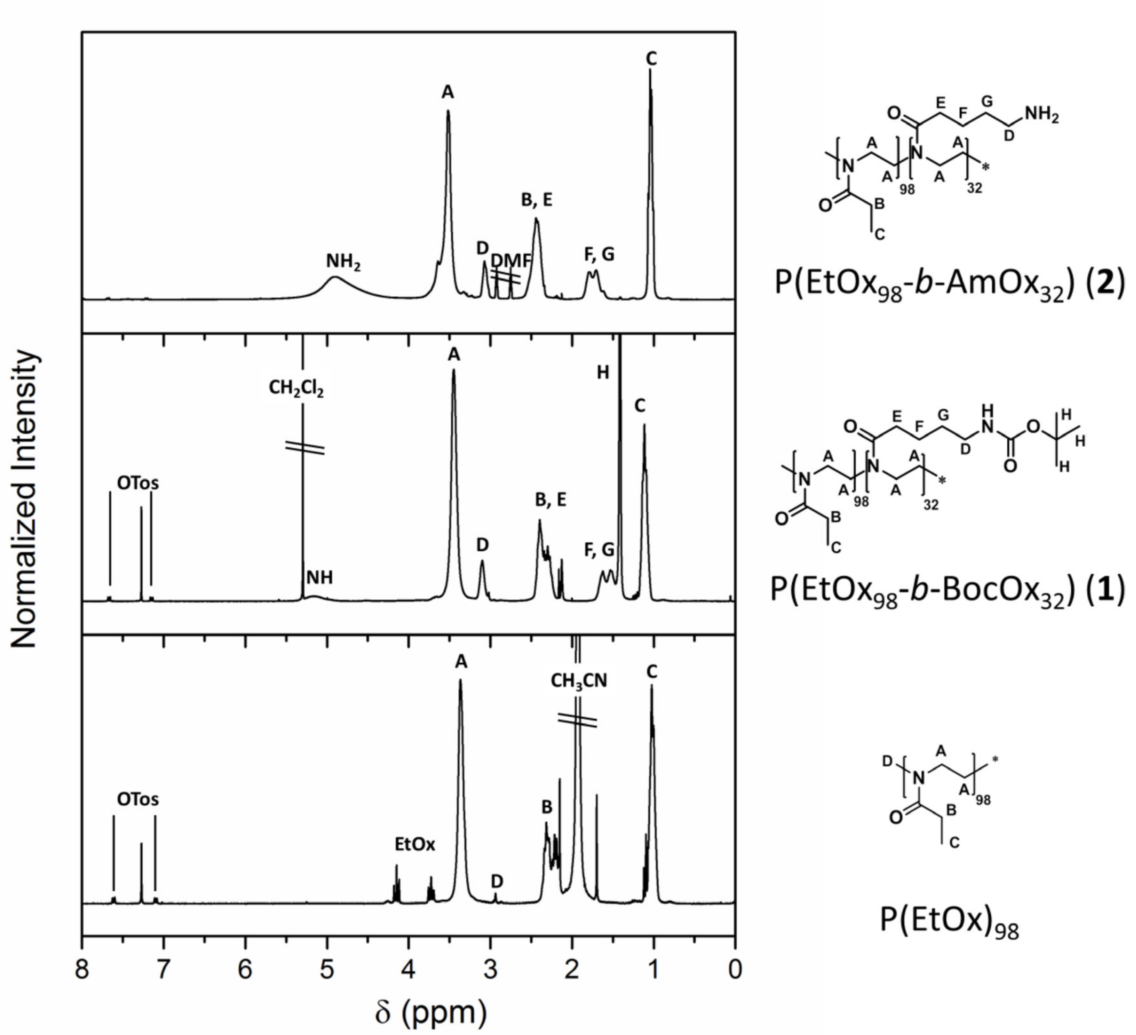

Supplementary Figure 1: ^{1}H-NMR spectra (300 MHz, top: MeOD, middle and bottom: $CDCl_3$) of poly(2-oxazoline) block copolymers.

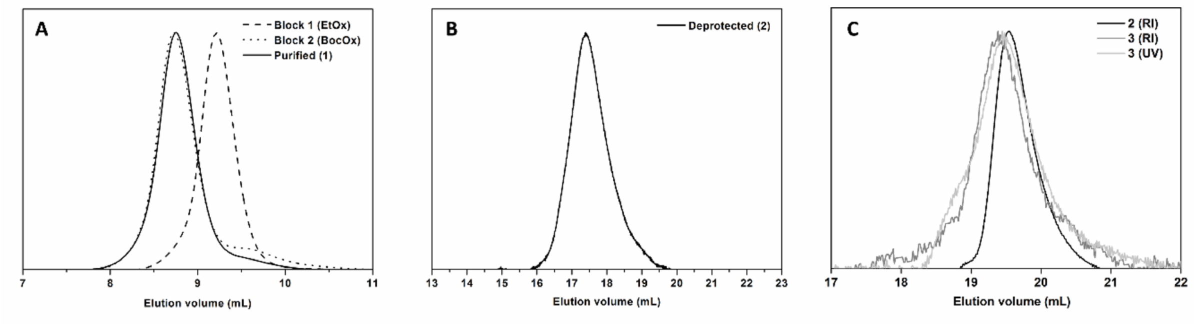

Supplementary Figure 2: SEC traces (DMAc, PS-cal.) of block copolymers before (**A**), and after (**B**) deprotection (in $CHCl_3$) as well as after labelling (**C**).

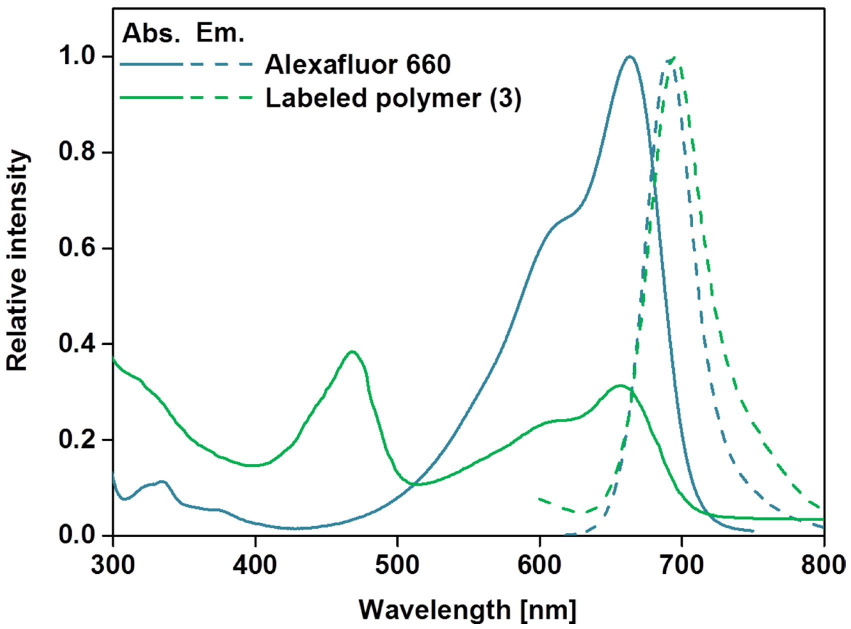

Supplementary Figure 3: Absorption and emission spectra (λ_{Ex} = 600 nm) of Alexafluor 660 and labeled poly(2-oxazoline) (3).

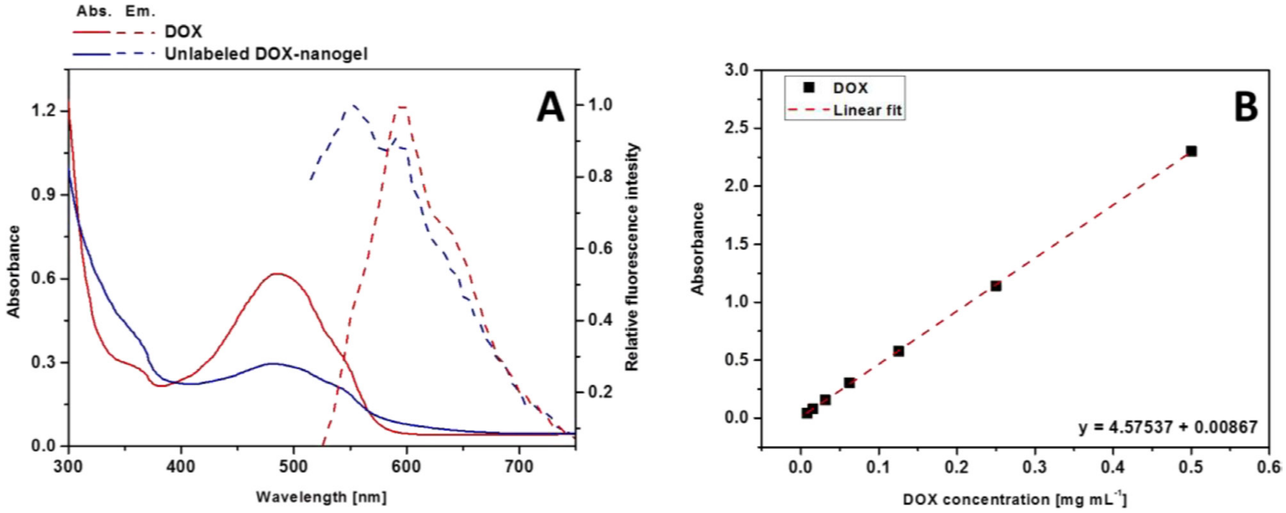

Supplementary Figure 4: (**A**) Absorption and emission spectra (excitation at λ = 450 nm) of DOX treated with a 10-fold excess of glutaraldehyde and the unlabeled DOX-nanogel. (**B**) Absorption calibration of DOX for the determination of nanogel loading efficiency.

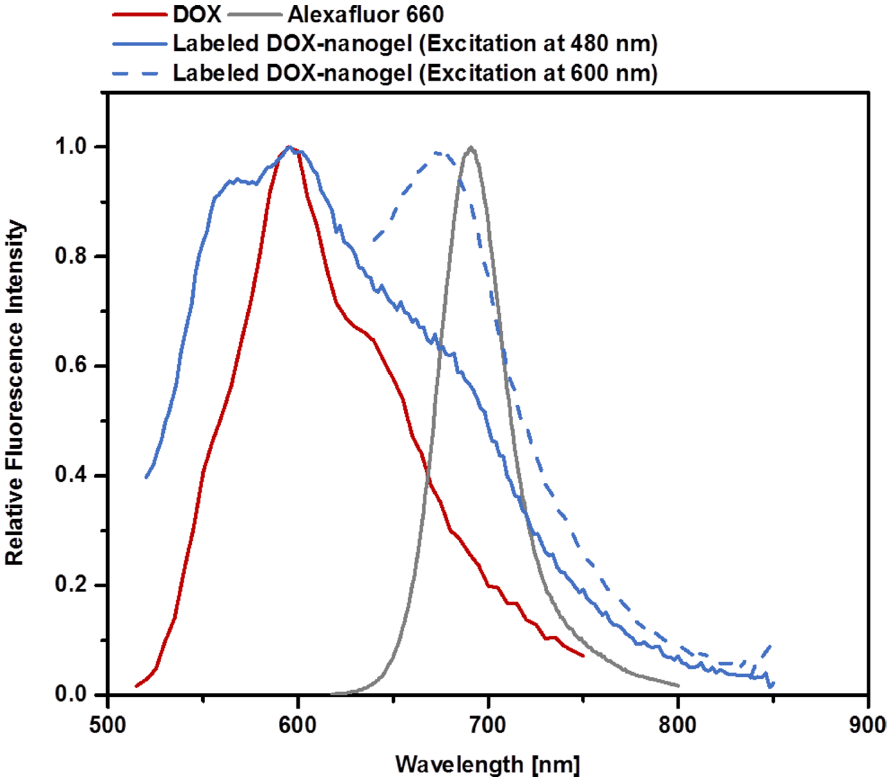

Supplementary Figure 5: Fluorescence spectra of DOX and the labeled DOX-nanogel excited at λ = 480 nm and Alexafluor 660 and the labeled DOX-nanogel excited at λ = 600 nm.

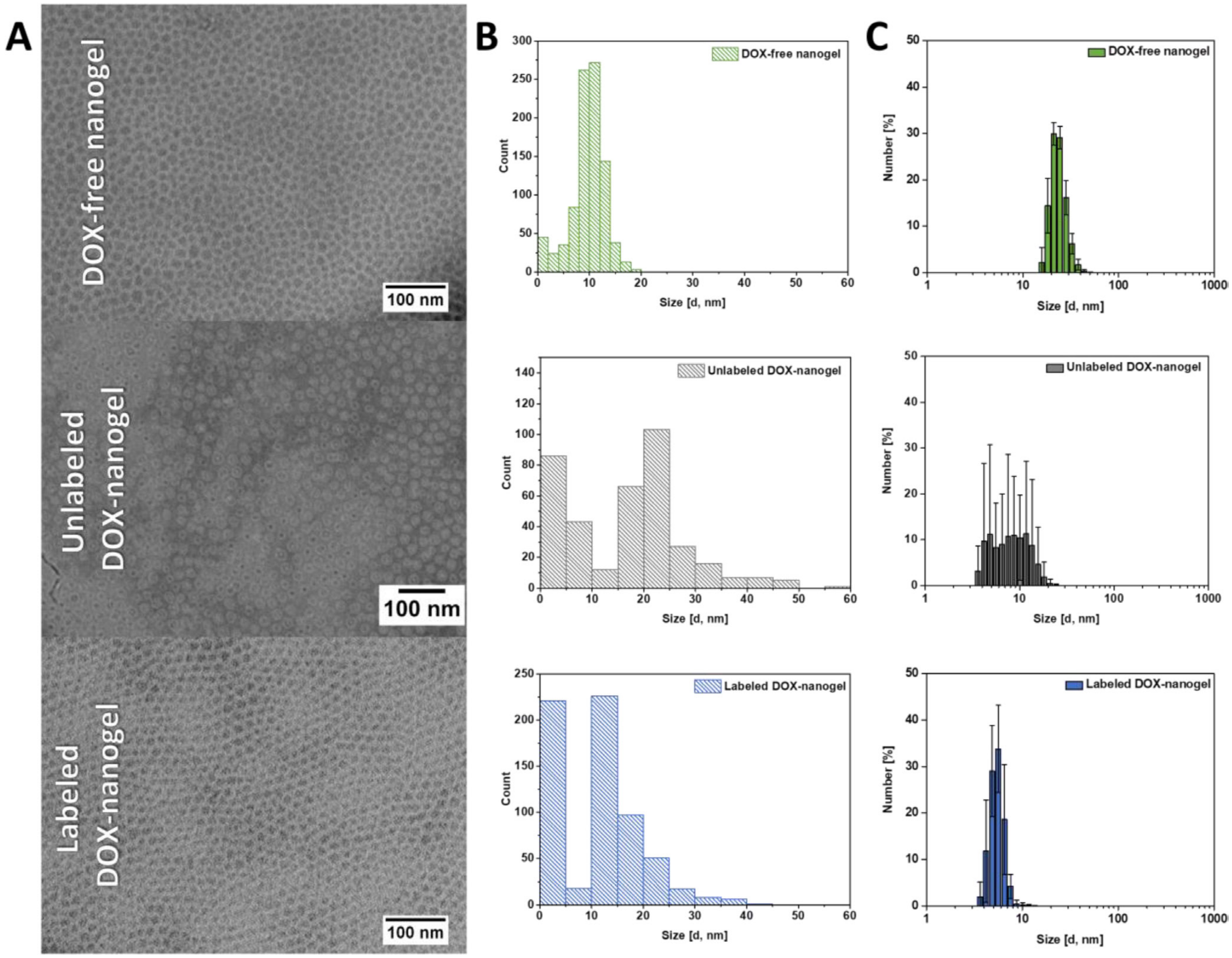

Supplementary Figure 6: (**A**) CryoTEM micrographs of DOX-free as well as labeled and unlabeled DOX-nanogels. (**B**) Size distribution histograms derived from cryoTEM image analysis using ImageJ. (**C**) Number weighted size distribution histograms as derived from Malvern Zetasizer software.

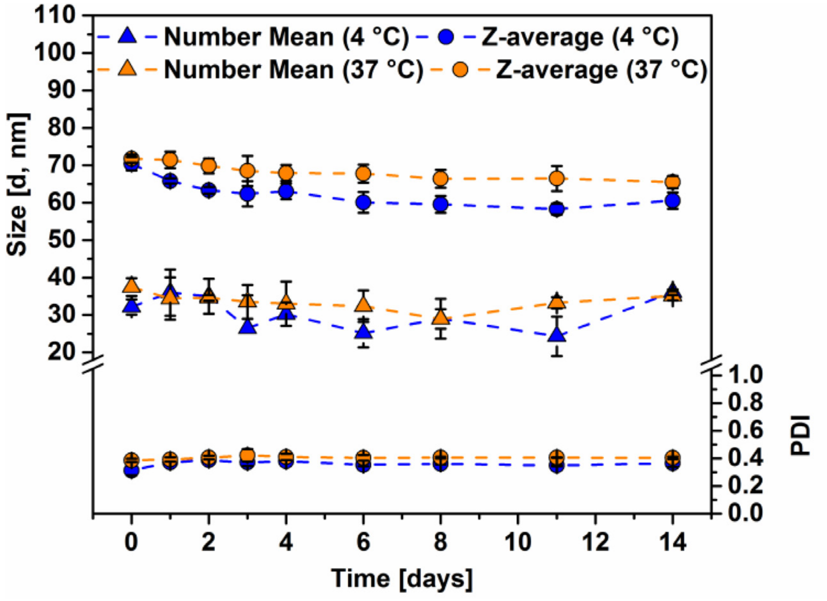

Supplementary Figure 7: Properties of labeled DOX-nanogels in 150 mM PBS (pH = 7.4) determined by DLS measurements. Nanogels were incubated at indicated temperatures for a certain time. Measurements were conducted at the indicated temperatures. PDI is derived from z-average.

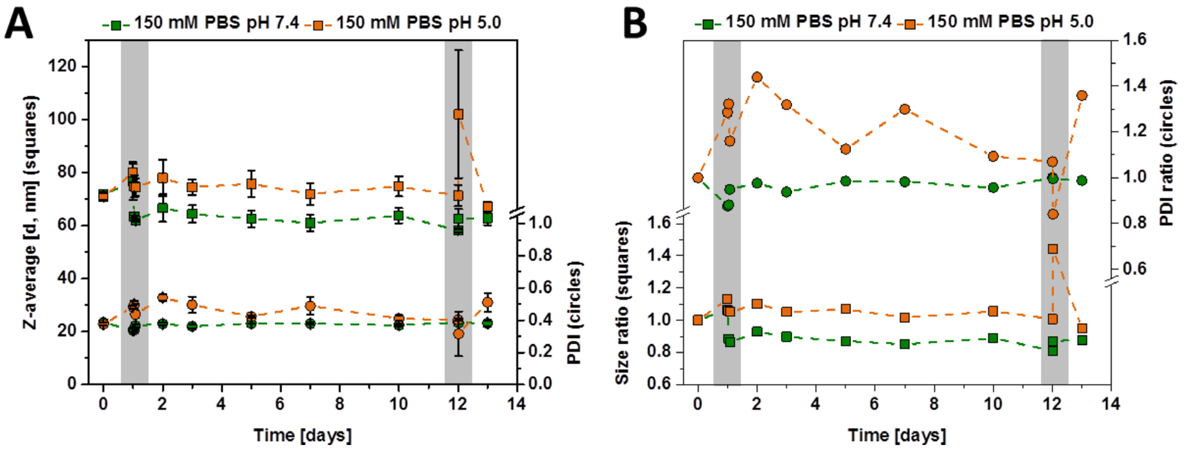

Supplementary Figure 8: Properties of labeled DOX-nanogels in 150 mM PBS (pH = 7.4 or 5.0) determined by DLS measurements. Nanogels were incubated at 37° C for a certain time. Measurements were conducted at 37° C. Grey boxes indicate time points of the addition of 100 mmol Glycine. (**A**) Actual values obtained by DLS measurements (*n* = 3, three measurements each). (**B**) Size and PDI ratios calculated by division of the value obtained on a certain day by the initial value (day 0).

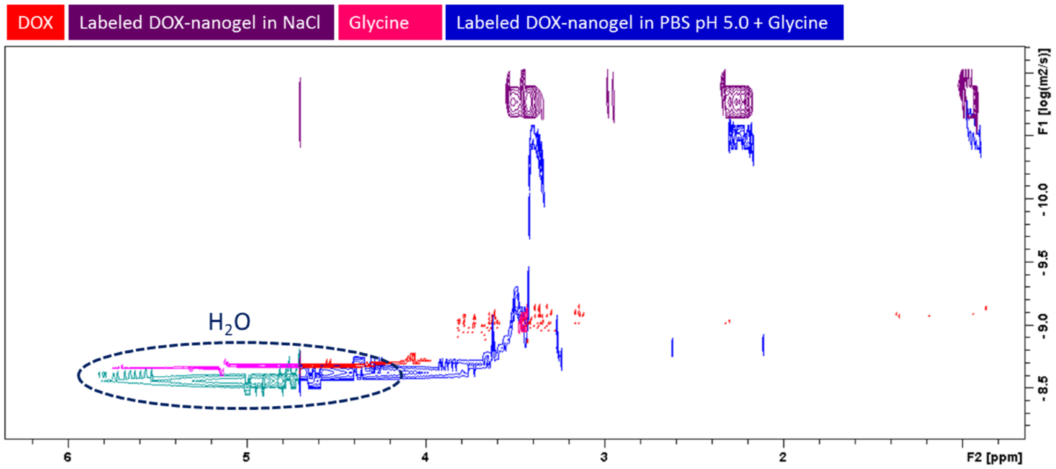

Supplementary Figure 9: Spectra of DOSY NMR (400 MHz, D2O) data of DOX in NaCl (red), the labeled DOX-nanogel in NaCl (purple), glycine (pink), and the labeled DOX-nanogel in PBS (pH = 5.0) + glycine. Peak superimpositions indicate similar diffusion coefficients of substances. Encircled area denotes water peak caused by the buffer. Peak overlays indicate similar diffusion coefficients of substances caused by the molar mass.

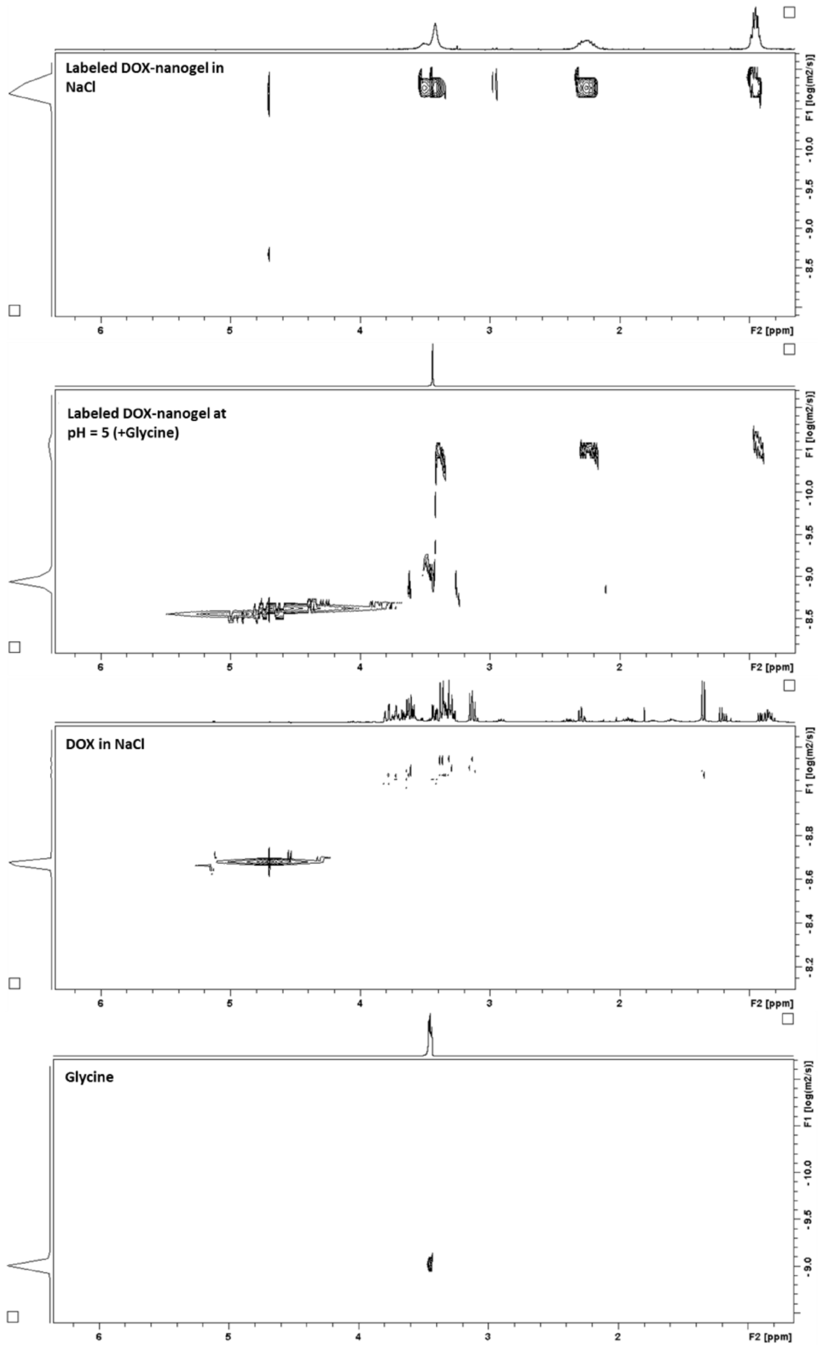

Supplementary Figure 10: Spectra of DOSY NMR (400 MHz, D_2O) data of the labeled DOX-loaded nanogel in PBS pH 5.0 + glycine, the labeled DOX-loaded nanogel in NaCl, DOX in NaCl and glycine (top to bottom).

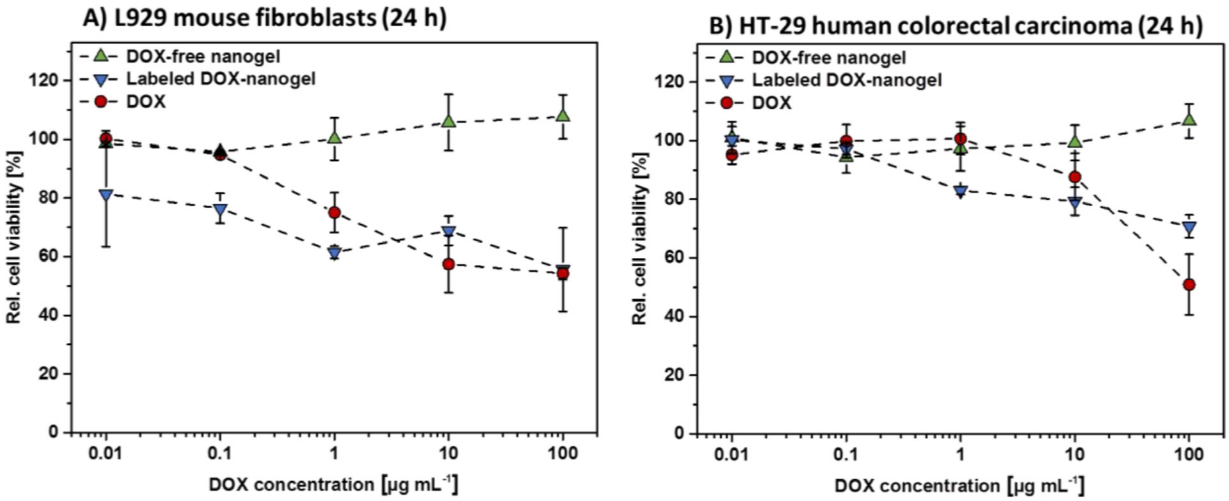

Supplementary Figure 11: Cytotoxicity of DOX-free nanogels, labeled DOX-nanogels as well as free DOX were determined by XTT assay. L292 mouse fibroblasts (**A**) as well as HT-29 human colorectal carcinoma cells (**B**) were incubated for 24 h. DOX loaded nanogels were used at a concentration where the amount of loaded drug resembles the amount of DOX used per data point (polymer concentration 17 times higher than DOX concentration). DOX-free nanogels were used at the same polymer concentration as labeled DOX-nanogels. Data are expressed as mean ± SD of six determinations.

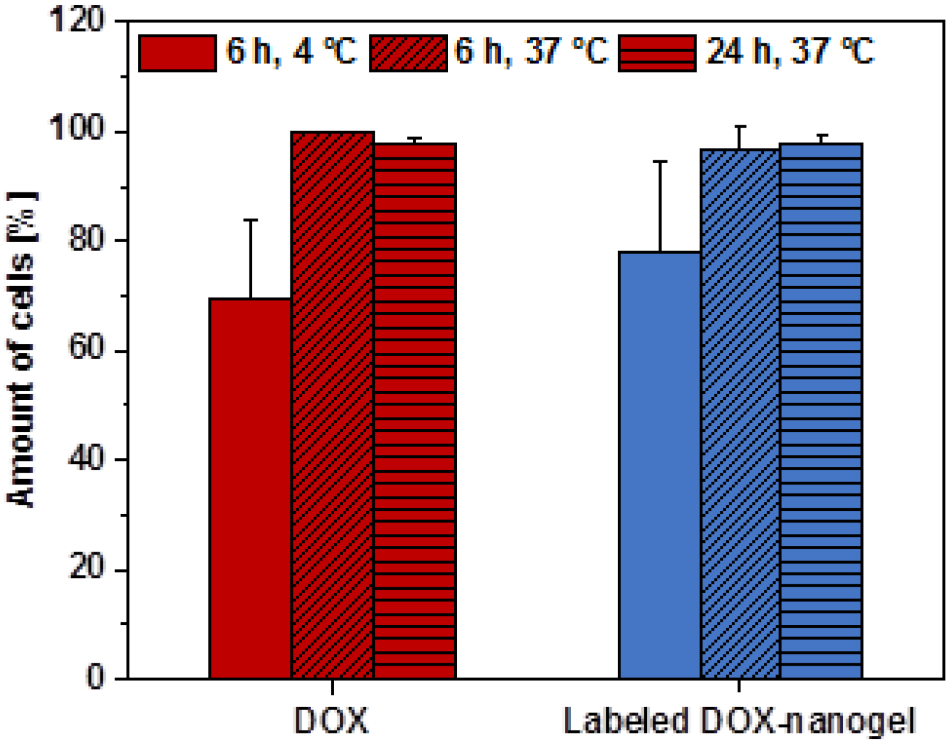

Supplementary Figure 12: Uptake of DOX and labeled DOX-nanogels into HT-29 cells (0.01 mg mL⁻¹) in dependence on the cultivation time and temperature. For MFI see Figure 3.

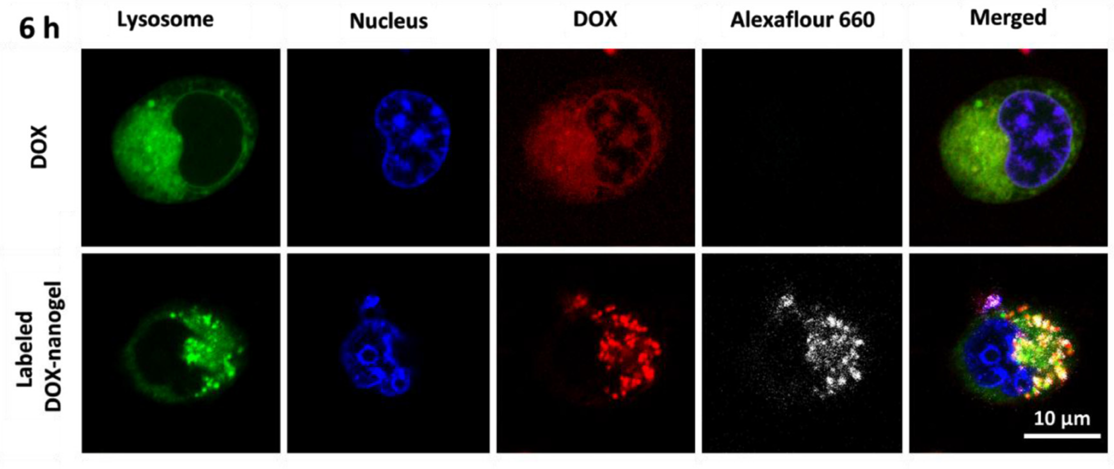

Supplementary Figure 13: CLSM images of free DOX as well as the labeled DOX-nanogels incubated with L929 mouse fibroblasts for 6 h. Lysosomal cellular compartments were stained green using LysoTracker Green DND-26 and the nucleus was labeled with Hoechst 33342 (blue). The fluorescence of DOX is depicted in red and the Alexafluor label of the polymer is shown in white.

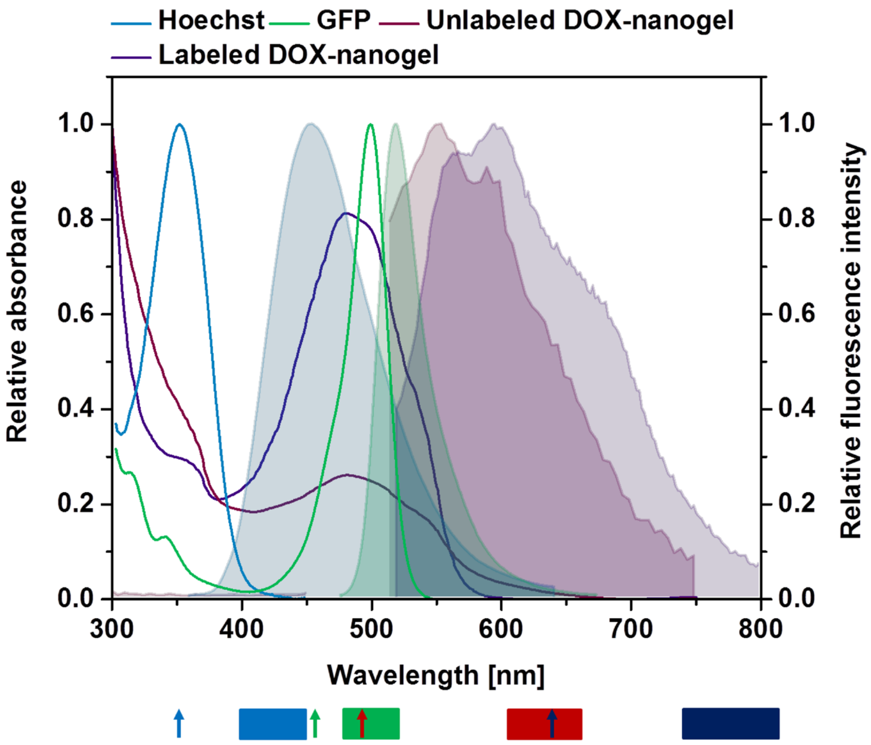

Channel	Name	Excitation [nm]	Emission [nm]
1	Hoechst	355	400 to 500
2	LysGFP	458	475 to 515
3	DOX	488	600 to 650
4	Polymer	633	725 to 800

Supplementary Figure 14: Absorption and emission spectra of dyes and compounds used in colocalization studies by CLSM.

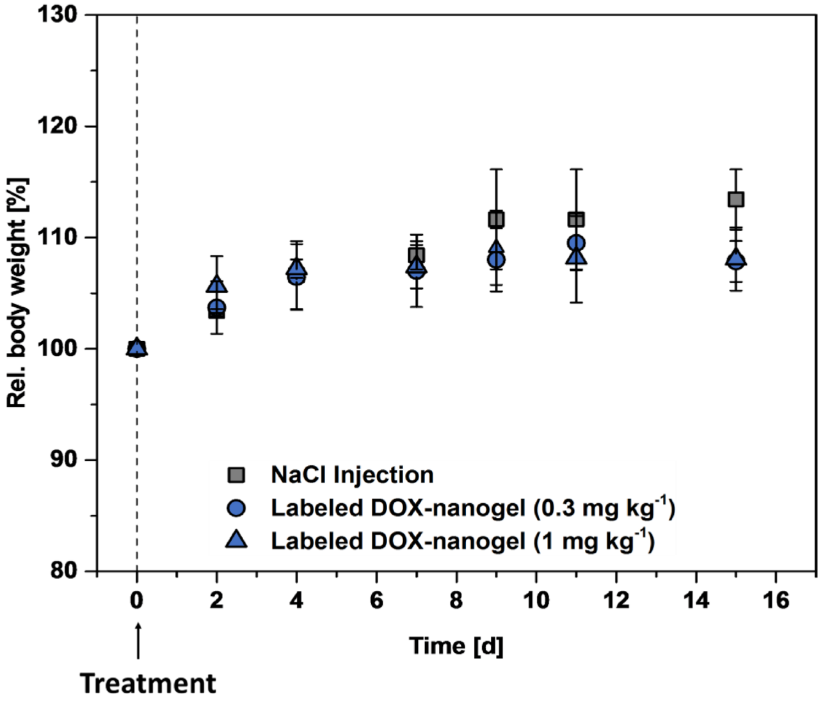

Supplementary Figure 15: Body weight development of male athymic nude mice after injection (dashed line) of a solution of labeled DOX-nanogel (corresponding to a DOX concentration of 0.3 or 1 mg kg^{-1}) dissolved in a 0.9 wt% NaCl solution or with the same volume of the 0.9 wt% NaCl solution as the negative control. Data are expressed as mean ± SD of 4 mice per group.

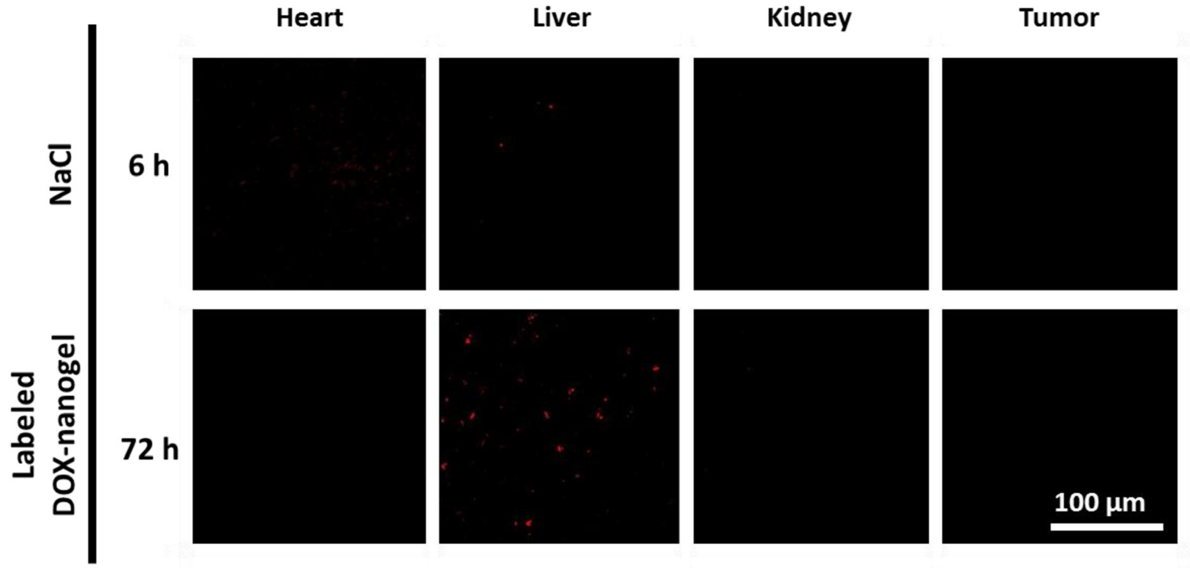

Supplementary Figure 16: Confocal fluorescence images of histological samples derived from organs of mice that were treated with either labeled DOX-nanogel (6) at 1 mg kg^{-1} or the same volume of a solution of 0.9 wt% NaCl in water. Fluorescence of DOX is shown in red. See Figure 7 for 6 h and 24 h samples of labeled DOX-nanogels (6).

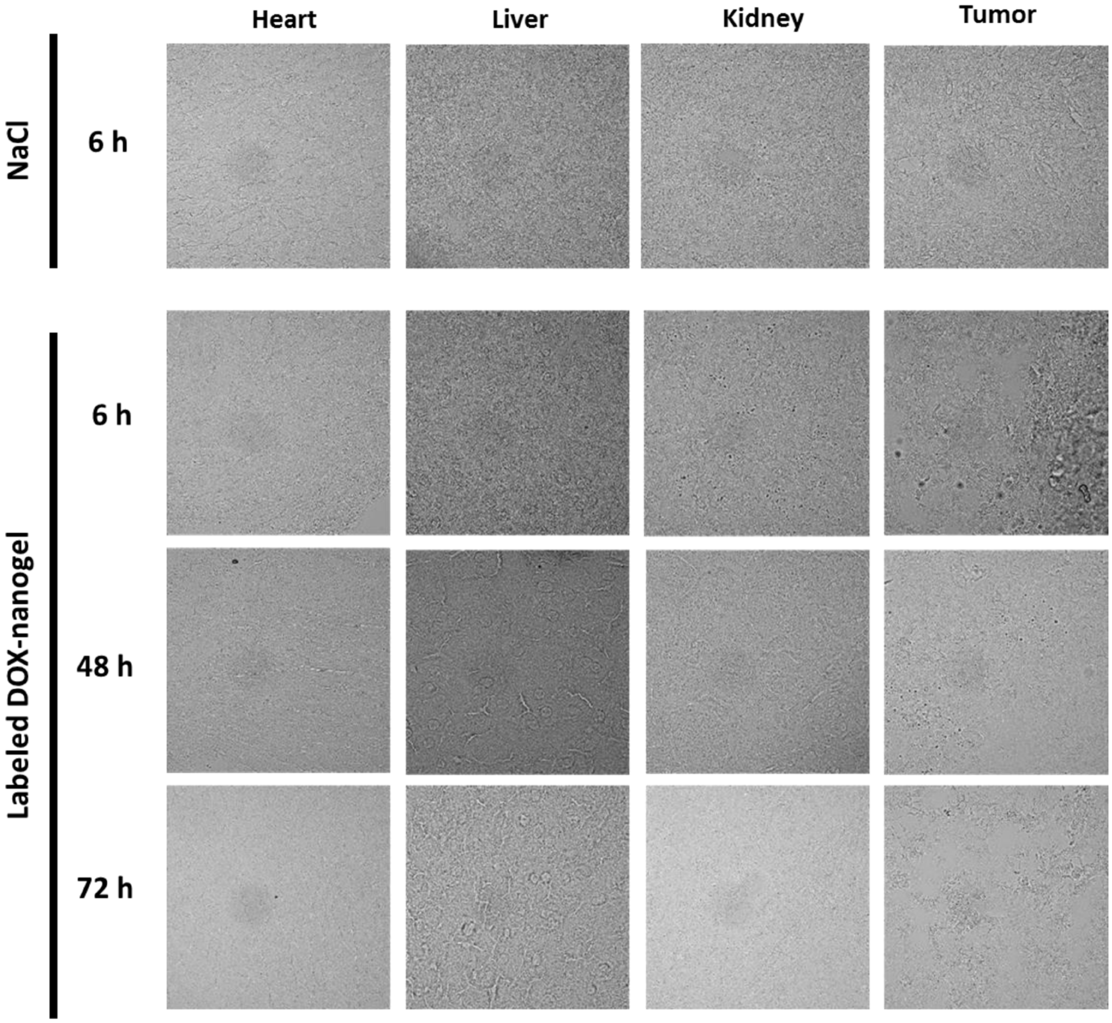

Supplementary Figure 17: Transmitted light CLSM measurements of histological samples derived from organs of mice that were treated with either labeled DOX-nanogel at 1 mg kg^{-1} or the same volume of a solution of 0.9 wt% NaCl in water. See Figure 7 for fluorescence pictures.

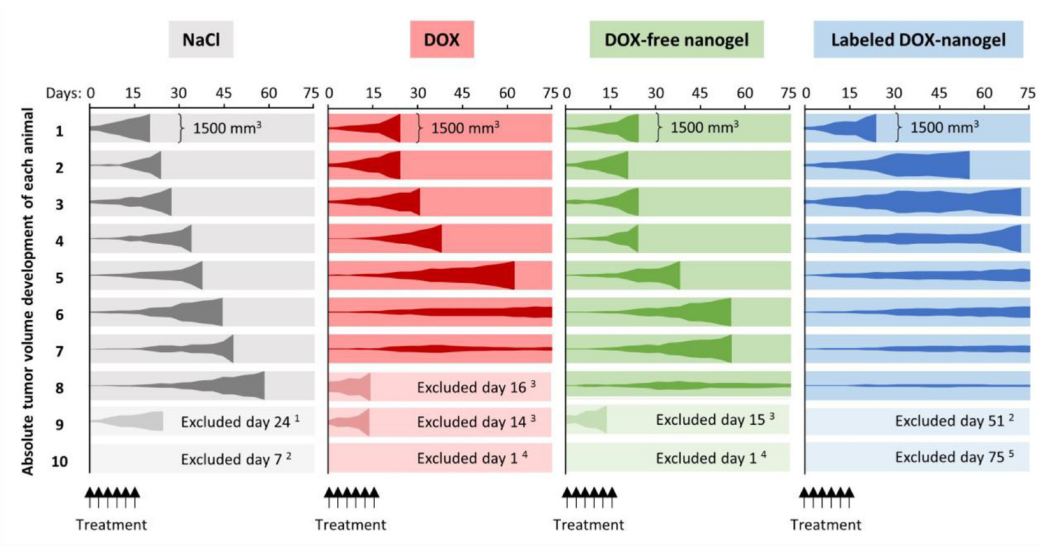

Supplementary Figure 18: Development of absolute tumor volume for each animal is illustrated over time from the beginning of treatment (day 0) until the maximum tumor volume of 1500 mm^3 was reached. Male nude mice were subcutaneously injected with HT-29 cells. When tumors reached a volume of 100 to 200 mm^3, mice received six doses of 0.9 wt% NaCl, DOX (1 mg kg-1), DOX-free nanogel and labeled DOX-nanogel (corresponding to 1 mg kg^{-1} DOX) via tail vein injection from day 0 to day 15. Individual animals were excluded from the study due to several reasons: [1]Termination because of infected wound, [2]Termination because of weight loss > 15%, [3]Termination because of maximum tumor volume before last injection, [4]Deceased during injection, 5 No palpable tumor.

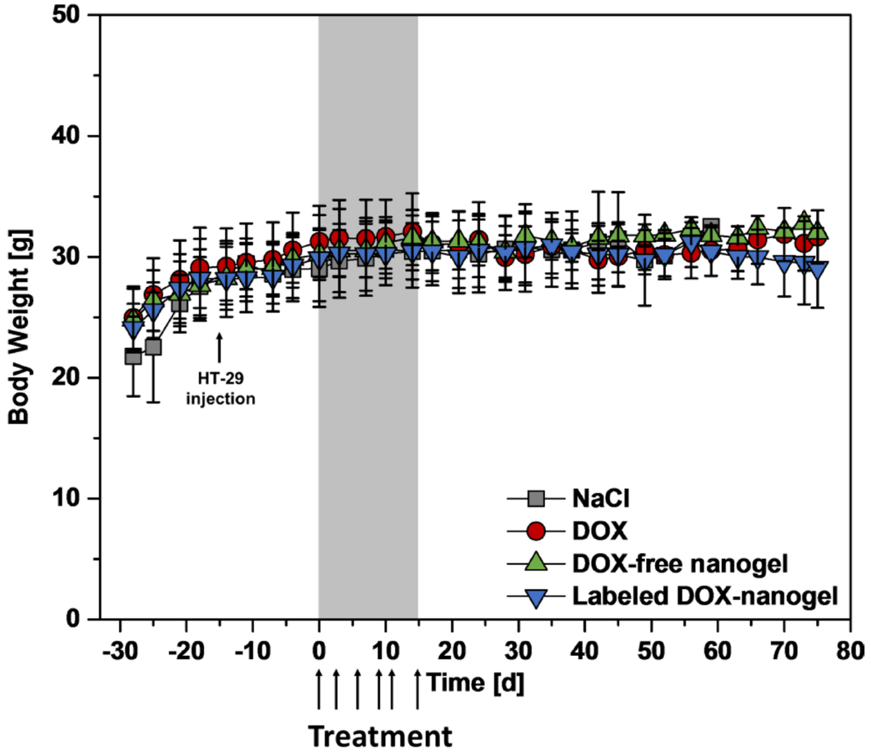

Supplementary Figure 19: Body weight development of male athymic nude mice over time from the beginning of the survival study until the maximum tumour volume of 1500 mm^3 was reached. Mice bearing HT-29 derived tumors received 6 doses of 0.9 wt% NaCl, DOX (1 mg kg^{-1}), DOX-free nanogel and labeled DOX-nanogel (corresponding to 1 mg kg^{-1} DOX) via tail vein injection from day 0 to day 15 (shown as grey area). Data are expressed as mean ± SD of 7–8 mice per group.
